# Effects of Rootstock, Pre-Grafting Light Intensity and Healing Relative Humidity on Photosynthetic Performance of Grafted Tomato Seedlings

**DOI:** 10.3390/plants15213300

**Published:** 2026-10-26

**Authors:** Tanzila Rafique, Kristina Laužikė

**Affiliations:** Lithuanian Research Centre for Agriculture and Forestry, Institute of Horticulture, Kauno Str. 30, LT-54333 Babtai, Kaunas District, Lithuania; kristina.lauzike@lammc.lt

**Keywords:** grafted tomato seedlings, rootstock, light intensity, relative humidity, graft success, photosynthesis, chlorophyll fluorescence

## Abstract

The production of high-quality grafted tomato seedlings depends on both the physiological condition of seedlings before grafting and the healing environment after grafting. The present study evaluated the combined effect of rootstocks, pre-grafting light intensity and healing relative humidity on graft success, growth, gas exchange, and chlorophyll fluorescence responses of grafted tomato seedlings. The tomato scion cultivar ‘Jalila F1’ was grafted onto four rootstocks: ‘Auroch’, ‘Ficus’, ‘Goldrake’, and ‘TOR23901’. Rootstock seedlings were grown under three pre-grafting light intensities (150, 250, and 350 µmol m^−2^·s^−1^) and, after grafting, seedlings were healed under three relative humidity levels (60%, 75% and 90% RH). Three-way ANOVA revealed significant effects of rootstock, light intensity, RH, and their interactions on most growth, photosynthetic, and chlorophyll fluorescence traits. Graft success rate varied among treatments, with the highest values observed at 250 µmol m^−2^·s^−1^ + 75% RH and 350 µmol m^−2^·s^−1^ + 60–75% RH. In contrast, 90% healing RH resulted in more variable graft success, particularly in ‘Ficus’. Rootstocks strongly influenced post-grafting growth, with ‘Auroch’ showing greater dry matter accumulation, whereas ‘Ficus’ produced thicker stems and larger leaf area. Gas-exchange responses indicated that 250 µmol m^−2^·s^−1^ pre-grafting light combined with 75% RH enhanced photosynthetic performance after healing. Chlorophyll fluorescence responses further showed treatment-dependent variation in the effective quantum yield of PSII [Y(II)], non-photochemical quenching (NPQ), and electron transport rate (ETR). Among the tested levels of pre-grafting PPFD and healing RH, 250 µmol m^−2^·s^−1^ combined with 75% healing relative humidity provided the most consistent overall performance in terms of graft success, seedling growth, and photosynthetic performance., although the magnitude of the response varied among rootstock genotypes.

## 1. Introduction

Tomato (*Solanum lycopersicum* L.) is one of the most important horticultural crops globally because of its high nutritional and economic value [1]. However, tomato productivity is increasingly constrained by biotic and abiotic stresses, including soil-borne pathogens, drought, salinity, and extreme temperatures. Grafting is widely used in tomato production to mitigate these limitations by combining a desirable scion with a vigorous or stress-tolerant rootstock [2,3,4]. However, the performance of grafted plants depends not only on rootstock–scion compatibility but also on the physiological quality of grafted seedlings produced during the nursery stage. The production of high-quality vegetable seedlings is crucial for successful crop production, as they directly influence plant growth and productivity. Using optimal seedlings can reduce transplant shock, enhance vegetative growth, and accelerate fruit maturation [5,6].

Graft healing is a critical phase in the grafting process, during which vascular connectivity is re-established between the scion and the rootstock [7,8]. Successful grafting requires suitable environmental conditions during healing and acclimatization to promote graft-union formation and restore physiological recovery between rootstock and scion [9,10]. Fluctuations in environmental factors can have dramatic effects on the quality and quantity of grafted seedling production. During this period, water and nutrient transport to the scion are limited, making the grafted seedling highly sensitive to dehydration, wilting, and reduced photosynthetic activity [11]. Relative humidity is therefore essential for maintaining tissue hydration and reducing water loss through transpiration during the early healing period, supporting graft-union formation and subsequent vascular reconnection [12]. However, the optimum humidity regime during healing remains debated. Buajaila et al. [13] reported improved graft survival under very high relative humidity (~100%) particularly when combined with moderate light levels, whereas Vu et al. [14] found that maintaining 90% RH for only the first 2–3 d after grafting, followed by a reduction to 70% RH, improved both graft-take and seedling quality whereas continuous exposure to 90% RH reduced seedling performance. Similarly, Masterson et al. (2016) [15] suggested that RH levels above 85% may not be necessary for successful tomato graft healing. These contrasting findings indicate that healing RH should be carefully regulated rather than maintained continuously at very high levels. Prolonged exposure to high humidity may become detrimental as the persistent moist environment increases the growth of bacterial and fungal pathogens, significantly increasing the risk of physiological disorders or disease at the vulnerable graft union and reduced seedling quality [16].

In addition to healing relative humidity, pre-grafting light intensity is another key environmental factor influencing grafted tomato performance because it determines the morphological and physiological condition of rootstocks and scions before grafting. In tomato, light regulates photosynthetic carbon assimilation, stomatal conductance, transpiration, chlorophyll fluorescence, biomass accumulation, and overall physiological efficiency [17]. Accordingly, appropriate light conditions during rootstock production are particularly important, as seedling vigor and quality strongly influence graft success and subsequent scion growth [18]. Recent work on commercial tomato rootstocks showed that increasing light intensity from 150 to 250 µmol m^−2^·s^−1^ improved growth and biomass accumulation, whereas a further increase to 350 µmol m^−2^·s^−1^ did not consistently enhance growth, with clear genotype-dependent responses [17]. During graft healing, light quality also affects seedling recovery: regulating the red:blue light ratio and supplementing green light improved tomato graft healing efficiency [19], and red LEDs containing 11–24% blue light during healing produced seedlings with greater stem diameter, root development and tolerance to transplant shock than fluorescent, monochromatic red or blue light [20]. In this way, light should be considered not merely as a background cultivation factor, but as an environmental modulator of rootstock–scion interactions [21]. Research studies on tomato seedlings for mechanical grafting reported that regulated light intensity and photoperiod affected hypocotyl growth, stem development, mechanical traits, and grafting suitability [12]. Similarly, studies on grafted tomato seedlings under artificial light or different LED spectra showed that light environment can modify seedling morphology, dry matter accumulation, photosynthetic performance, chlorophyll fluorescence, and recovery after grafting [22]. Previous studies have also shown that photoperiod and LED light quality can influence growth, physiological disorders, and photosynthetic performance of grafted tomato seedlings during healing and protected cultivation [23,24].

Pre-grafting light and healing relative humidity may be particularly important when considered together. Pre-grafting light determines the morphological and physiological status of seedlings entering the healing phase, whereas healing relative humidity subsequently influences water loss and physiological recovery during graft-union establishment. Previous work has shown that tomato rootstocks differ in their growth and photosynthetic responses to pre-grafting light intensity [17]; however, it remains unclear whether these rootstock-dependent responses are further influenced by healing relative humidity. Although light and humidity have individually been recognized as important environmental factors during graft production, information on their combined effects across different tomato rootstocks remains limited. Therefore, the present study aimed to investigate the combined effects of pre-grafting light intensity and healing relative humidity on grafted tomato seedlings and to identify suitable environmental combinations for producing high-quality grafted seedlings. We hypothesized that the effects of pre-grafting light intensity and healing relative humidity on graft success, growth, and physiological performance would vary among rootstock genotypes, resulting in significant rootstock × light intensity × healing RH interactions.

## 2. Results

### 2.1. Graft Success

**Figure 1:**
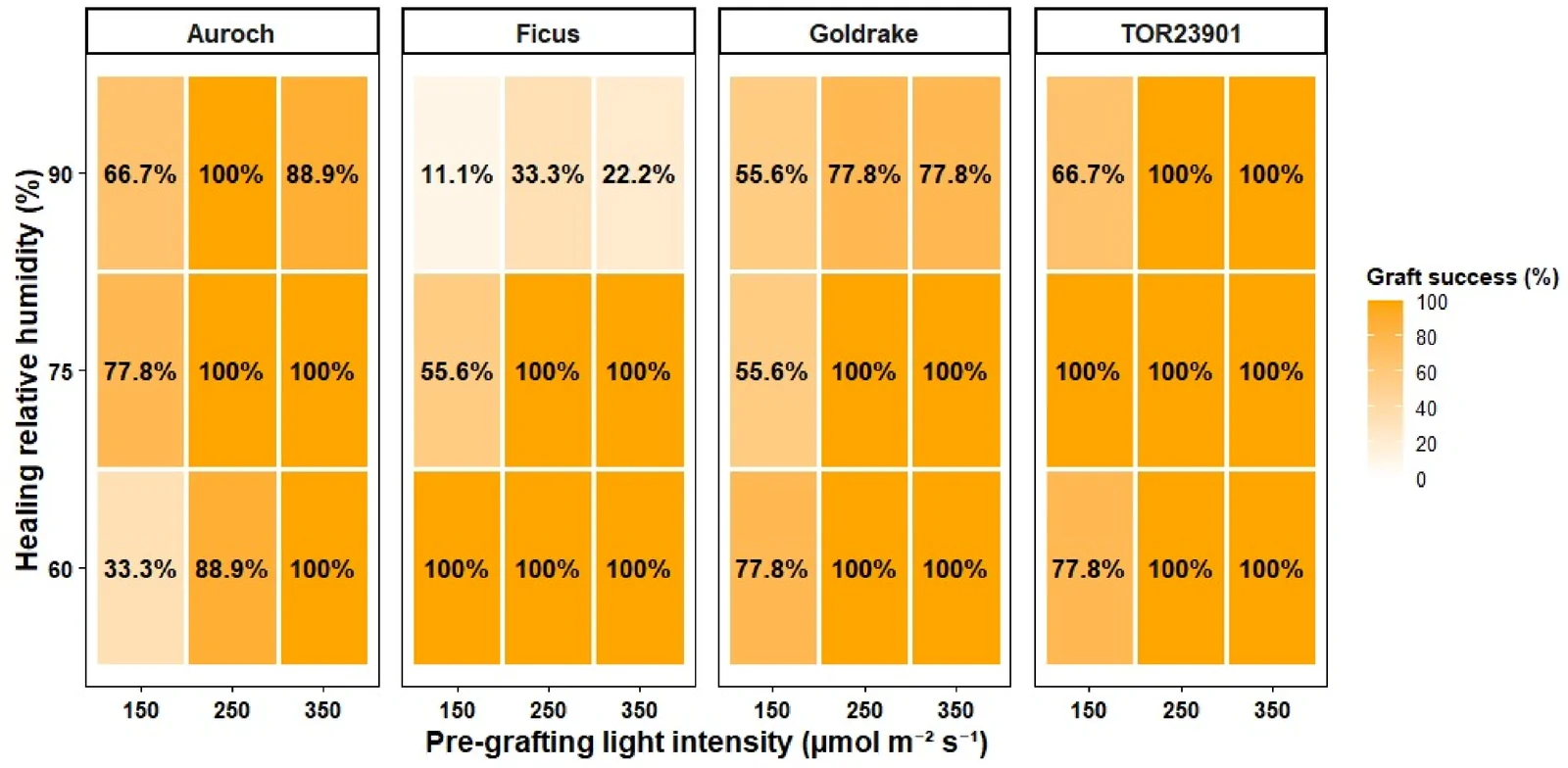
Graft success of tomato seedlings in response to rootstock, pre-grafting light intensity, and healing relative humidity (RH). Values represent the percentage of successfully healed grafts out of nine grafted plants per treatment. Graft-success data are presented descriptively, without inferential statistical comparison.

Graft success varied among rootstocks, pre-grafting light intensity, and different humidity levels (Figure 1). Graft success was inconsistent among rootstocks at 150 µmol m^−2^·s^−1^, with lower survival especially in ‘Ficus’ at 90% RH, while ‘TOR23901’ maintained high graft success at 75% RH. For a light intensity of 250 µmol m^−2^·s^−1^, graft success improved, and the combination of 250 µmol m^−2^·s^−1^ with 75% RH resulted in 100% graft success among all rootstocks. At a light intensity of 350 µmol m^−2^·s^−1^, both 60% and 75% RH resulted in 100% graft success among all rootstocks, whereas 90% RH reduced graft success in ‘Auroch’, ‘Goldrake’ and ‘Ficus’. Overall, graft success was highest and most consistent at 250–350 µmol m^−2^·s^−1^ pre-grafting light combined with 75% RH, while 90% RH resulted in more variable responses, particularly in ‘Ficus’.

### 2.2. Growth Parameters

**Figure 2:**
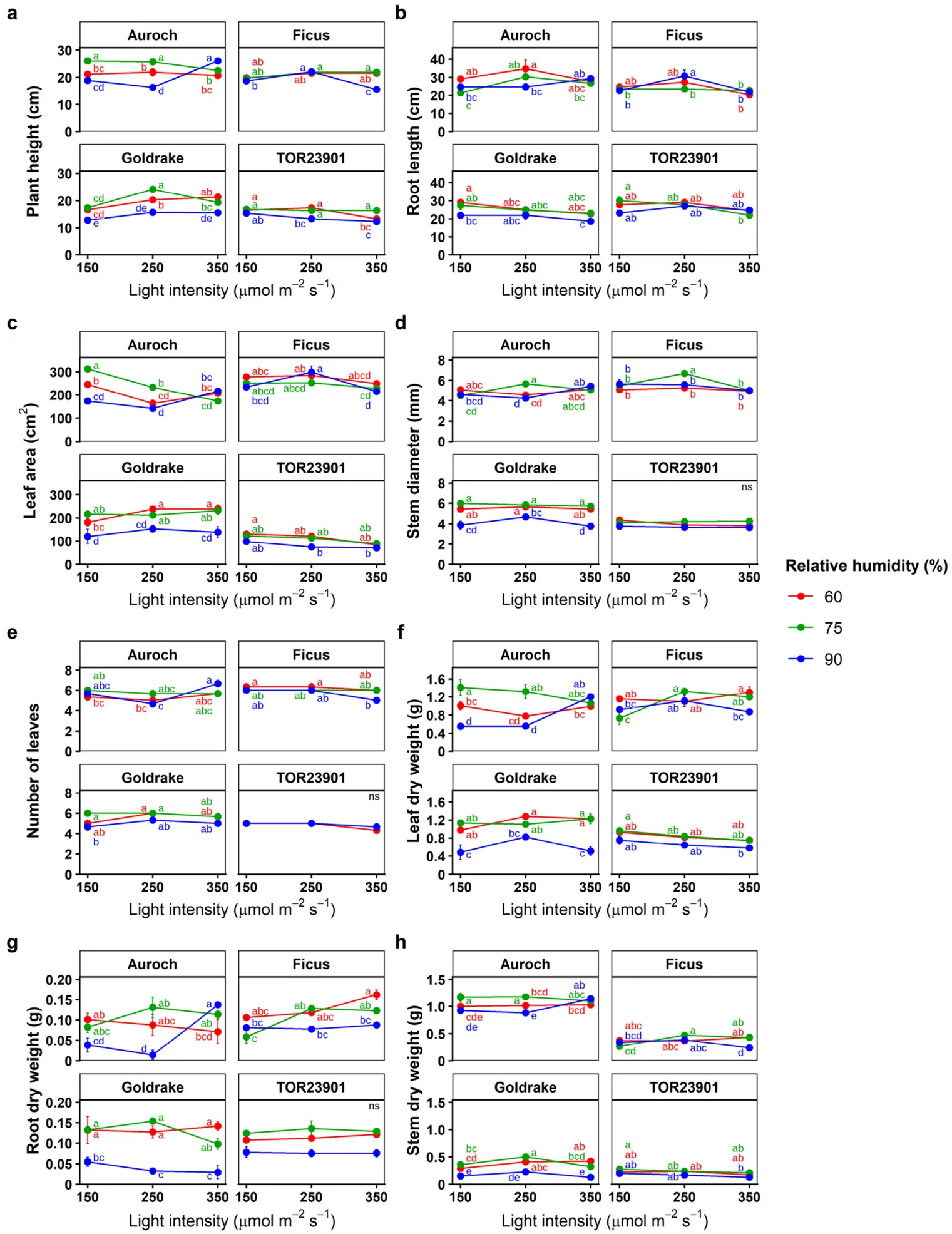
Interactive effects of pre-grafting light intensity and healing relative humidity (RH) on growth and biomass traits of grafted tomato seedlings using four rootstocks (‘Auroch’, ‘Ficus’, ‘Goldrake’, and ‘TOR23901’): (**a**) plant height, (**b**) root length, (**c**) leaf area, (**d**) stem diameter, (**e**) number of leaves, (**f**) leaf dry weight, (**g**) root dry weight, and (**h**) stem dry weight. Data are presented as means (*n* = 3), with error bars representing standard error. Within each rootstock, different lowercase letters indicate significant differences among light intensity × RH combinations according to Tukey’s HSD test (*p* < 0.05); ns, not significant. Colored lines represent healing RH levels of 60, 75, and 90% across pre-grafting light intensities of 150, 250, and 350 µmol m^−2^·s^−1^. Lines connect treatment means to aid visual comparison and do not imply continuous responses between treatment levels.

The varying rootstocks, light intensities, humidity levels, and their interactions significantly affected the biometric indicators, thus indicating that seedling quality after grafting depended on the combined effect of rootstock, light conditioning, and healing humidity. However, some exceptions existed e.g., light intensity x humidity interactions for root length, leaf number, and dry weight were non-significant. Mean values and the significance of the main and interactive effects are presented in Table 1 and Table 2, respectively, while the rootstock-specific interaction patterns are shown in Figure 2.

Among rootstock treatments, seedlings grafted on ‘Auroch’ showed significantly greater plant height, root length, and stem dry weight, whereas seedlings grafted onto ‘Ficus’ produced the greatest leaf area, stem diameter, and dry weight of root and leaf. Seedlings grafted on ‘Goldrake’ showed a moderate response for most growth parameters, whereas seedlings grafted on TOR23901 showed lower plant height, leaf area, stem diameter, and stem and leaf dry weight.

The effect of pre-grafting light intensity differed among graft combinations. Among light intensities, 250 µmol m^−2^·s^−1^ showed the greatest plant height, root length, stem diameter, and stem dry weight compared with 150 and 350 µmol m^−2^·s^−1^. However, this response was not the same for all grafted seedlings, indicating a rootstock-dependent effect of pre-grafting light conditions. Seedlings grafted onto ‘Auroch’ showed higher plant height and stem diameter at 250 µmol m^−2^·s^−1^, particularly under 75% RH, whereas root length was greatest at the same light intensity combined with 60% RH. Seedlings grafted onto ‘Ficus’ also responded favorably to 250 µmol m^−2^·s^−1^ for leaf area and stem diameter. In ‘Goldrake’, plant height and stem diameter were higher at 250 µmol m^−2^·s^−1^ under 75% RH, whereas ‘TOR23901’ showed variable responses across growth traits and humidity levels.

Healing relative humidity also modified the growth response of grafted tomato seedlings. Overall, 75% relative humidity produced significantly greater plant height, stem diameter, leaf area, number of true leaves, root dry weight, stem dry weight, and leaf dry weight. In contrast, seedlings healed under 90% relative humidity showed reduced leaf area, stem diameter, and dry matter accumulation compared with 75% humidity. However, the response depends on the graft combination. Seedlings grafted onto ‘Auroch’ performed well under 75% RH, particularly under 150 and 250 µmol m^−2^·s^−1^, whereas seedlings grafted onto ‘Ficus’ showed strong leaf area and stem diameter responses under 250 µmol m^−2^·s^−1^ combined with 75% or 90% RH. In contrast, 90% RH generally reduced growth performance in ‘Goldrake’ and ‘TOR23901’, especially for plant height, leaf area, and stem diameter. This indicates that very high humidity did not improve seedling growth and its effect depends on the rootstock used.

Overall, ‘Auroch’ and ‘Ficus’ rootstocks resulted in stronger morphological scion performance after grafting. ‘Auroch’ showed greater seedling dry matter accumulation, particularly in stem and leaf tissues, whereas tomato grafted to ‘Ficus’ rootstock produced larger leaf area and thicker stems. Among the pre-grafting light treatments, 250 µmol m^−2^·s^−1^ supported better shoot development and biomass accumulation, while healing at 75% relative humidity resulted in better seedling growth than 60% and 90% relative humidity.

### 2.3. Photosynthetic Gas Exchange and PSII Efficiency

**Figure 3:**
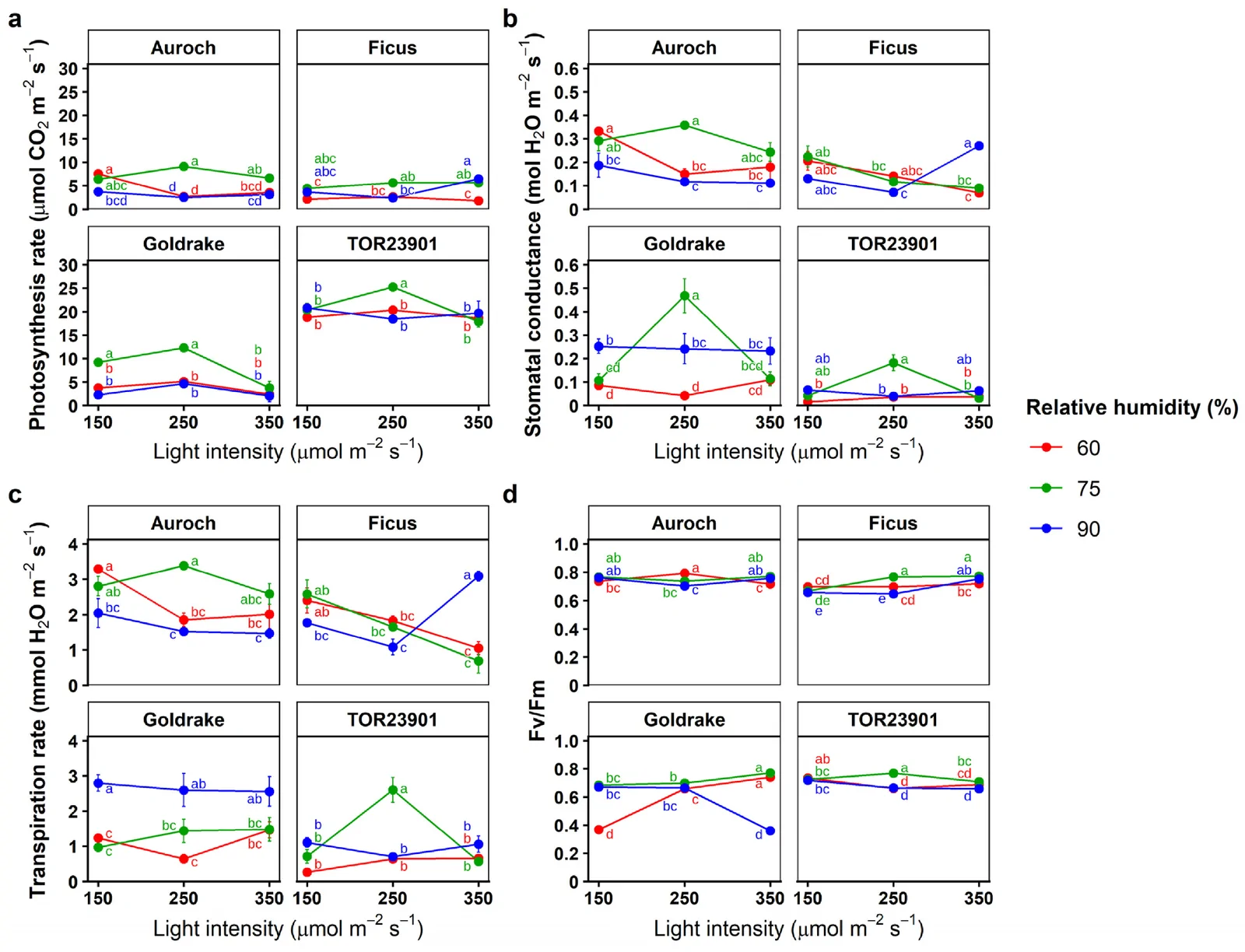
Interactive effects of pre-grafting light intensity and healing relative humidity (RH) on physiological traits of grafted tomato seedlings using four rootstocks (‘Auroch’, ‘Ficus’, ‘Goldrake’, and ‘TOR23901’): (**a**) photosynthetic rate (A), (**b**) stomatal conductance (gs), (**c**) transpiration rate (E), and (**d**) maximum quantum efficiency of PSII (Fv/Fm). Data are presented as means (*n* = 3), with error bars representing standard error. Within each rootstock, different lowercase letters indicate significant differences among light intensity × RH combinations according to Tukey’s HSD test (*p* < 0.05). Colored lines represent healing RH levels of 60, 75, and 90% across pre-grafting light intensities of 150, 250, and 350 µmol m^−2^·s^−1^. Lines connect treatment means to aid visual comparison and do not imply continuous responses between treatment levels.

Photosynthetic gas-exchange traits and PSII efficiency of grafted tomato seedlings were significantly affected by rootstock, pre-grafting light intensity, healing relative humidity, and their interactions (Table 2 and Table 3). The significant interaction effects indicate that the physiological response of grafted seedlings was not uniform across all graft combinations but depended on the specific rootstock and environmental conditions used during the preparation of seedlings and healing. The corresponding rootstock-specific interaction patterns are shown in Figure 3.

**Table 3:** Effects of rootstock, pre-grafting light intensity and healing relative humidity on gas exchange and chlorophyll fluorescence of grafted tomato seedlings.

Factor	Level	Net Photosynthetic Rate (µmol CO_2_ m^−2^·s^−1^)	Stomatal Conductance (mol H_2_O m^−2^·s^−1^)	Transpiration Rate (mmol H_2_O m^−2^·s^−1^)	Maximum Quantum Efficiency of PSII (Fv/Fm)
Rootstock	Auroch	5.02 ± 0.46 B	0.219 ± 0.020 A	2.33 ± 0.16 A	0.749 ± 0.006 A
	Ficus	3.86 ± 0.34 C	0.147 ± 0.015 B	1.79 ± 0.16 B	0.709 ± 0.009 B
	Goldrake	5.05 ± 0.67 B	0.183 ± 0.027 AB	1.68 ± 0.16 B	0.623 ± 0.028 C
	TOR23901	20.02 ± 0.52 A	0.057 ± 0.010 C	0.92 ± 0.13 C	0.701 ± 0.007 B
Light intensity (µmol m^−2^·s^−1^)	150	8.59 ± 1.17 A	0.162 ± 0.018 A	1.83 ± 0.17 A	0.682 ± 0.017 B
	250	9.25 ± 1.30 A	0.164 ± 0.023 A	1.66 ± 0.15 AB	0.704 ± 0.008 A
	350	7.62 ± 1.14 B	0.129 ± 0.015 B	1.55 ± 0.15 B	0.701 ± 0.018 A
Relative humidity (%)	60	7.44 ± 1.18 B	0.117 ± 0.016 C	1.44 ± 0.15 B	0.684 ± 0.017 B
	75	10.55 ± 1.15 A	0.189 ± 0.023 A	1.79 ± 0.17 A	0.736 ± 0.006 A
	90	7.48 ± 1.23 B	0.149 ± 0.015 B	1.82 ± 0.14 A	0.667 ± 0.017 C

Values are means ± SE (*n* = 27 for rootstock; *n* = 36 for light intensity and relative humidity; each observation represents one plant per replicate). Within each factor, means in a column followed by different uppercase letters differ significantly according to Tukey’s HSD test (*p* < 0.05). Because the rootstock × light intensity × relative humidity interaction was significant for all traits (Table 4), treatment combinations within each rootstock are compared in Figure 3 and Table S4.

**Table 4:** Analysis of variance (F-values and *p* values) for gas exchange and chlorophyll fluorescence parameters of grafted tomato seedlings.

Source of Variation	df	Net Photosynthetic Rate (µmol CO_2_ m^−2^·s^−1^)	Stomatal Conductance (mol H_2_O m^−2^·s^−1^)	Transpiration Rate (mmol H_2_O m^−2^·s^−1^)	Maximum Quantum Efficiency of PSII (Fv/Fm)
Rootstock (R)	3	961.06 (<0.001)	44.51 (<0.001)	46.23 (<0.001)	368.43 (<0.001)
Light intensity (LI)	2	14.52 (<0.001)	4.65 (0.013)	3.54 (0.034)	26.03 (<0.001)
Relative humidity (RH)	2	68.68 (<0.001)	15.98 (<0.001)	7.84 (<0.001)	227.14 (<0.001)
R × LI	6	9.67 (<0.001)	6.32 (<0.001)	5.24 (<0.001)	54.96 (<0.001)
R × RH	6	7.20 (<0.001)	10.42 (<0.001)	16.79 (<0.001)	45.63 (<0.001)
LI × RH	4	15.02 (<0.001)	16.18 (<0.001)	10.56 (<0.001)	94.75 (<0.001)
R × LI × RH	12	3.72 (<0.001)	5.32 (<0.001)	5.85 (<0.001)	146.20 (<0.001)
Error	72	–	–	–	–

Values are F-ratios with exact *p* values in parentheses from a three-way ANOVA with rootstock (R), pre-grafting light intensity (LI) and healing relative humidity (RH) as fixed factors (*n* = 3 replicates per treatment combination). df, degrees of freedom. Complete ANOVA results (SS, MS, F, P) are given in Table S2.

Net photosynthetic rate (A) differed among grafted tomato seedlings depending on the specific rootstock. Seedlings grafted onto ‘TOR23901’ showed the highest photosynthetic rate, whereas seedlings grafted onto ‘Ficus’ showed the lowest values of photosynthetic rate. Among pre-grafting light treatments, 250 µmol m^−2^·s^−1^ resulted in the highest photosynthetic activity, while 350 µmol m^−2^·s^−1^ reduced photosynthetic performance. Regarding healing relative humidity, seedlings that healed under 75% RH showed a higher photosynthetic rate than those healed under 60% or 90% RH.

Stomatal conductance (gs) also varied significantly among graft combinations. Seedlings grafted onto ‘Auroch’ showed the highest stomatal conductance, whereas seedlings grafted onto ‘TOR23901’ showed the lowest values. Regarding pre-grafting light intensities, higher stomatal conductance was recorded at 150 and 250 µmol m^−2^·s^−1^ than at 350 µmol m^−2^·s^−1^. Among healing humidity treatments, higher gs values were recorded at 75% RH, while lower stomatal conductance was observed at 60% and 90% RH.

Transpiration rate was also significantly influenced by rootstock, light intensity, and healing humidity. Seedlings grafted onto ‘Auroch’ showed the highest transpiration rate, whereas seedlings grafted onto ‘TOR23901’ showed the lowest values of transpiration rate. Higher transpiration was observed in seedlings grown under light intensities of 150 and 250 µmol m^−2^·s^−1^ before grafting and decreased under 350 µmol m^−2^·s^−1^. Among humidity treatments, the highest transpiration rate was recorded under 90% RH, although 75% RH also showed a higher photosynthetic rate and stomatal conductance.

The maximum quantum efficiency of photosystem II (PSII) varied significantly among treatments. Seedlings grafted onto ‘Auroch’ showed the highest Fv/Fm, whereas seedlings grafted onto ‘TOR23901’ showed the lowest values. Among light treatments, 250 and 350 µmol m^−2^·s^−1^ maintained higher Fv/Fm than 150 µmol m^−2^ s. Regarding the healing humidity, a significantly higher Fv/Fm was recorded for 75% relative humidity, whereas a decline in Fv/Fm was observed at 90% relative humidity indicating that too high humidity may have reduced photochemical performance.

Overall, 250 µmol m^−2^·s^−1^ pre-grafting light intensity combined with 75% RH healing conditions showed stronger photosynthetic performance and PSII efficiency in grafted tomato seedlings. Seedlings grafted onto ‘Auroch’ maintained higher stomatal conductance, transpiration, and Fv/Fm, whereas seedlings grafted onto ‘TOR23901’ showed the highest photosynthetic rate but lower photochemical efficiency.

### 2.4. Chlorophyll Fluorescence Parameters

**Figure 4:**
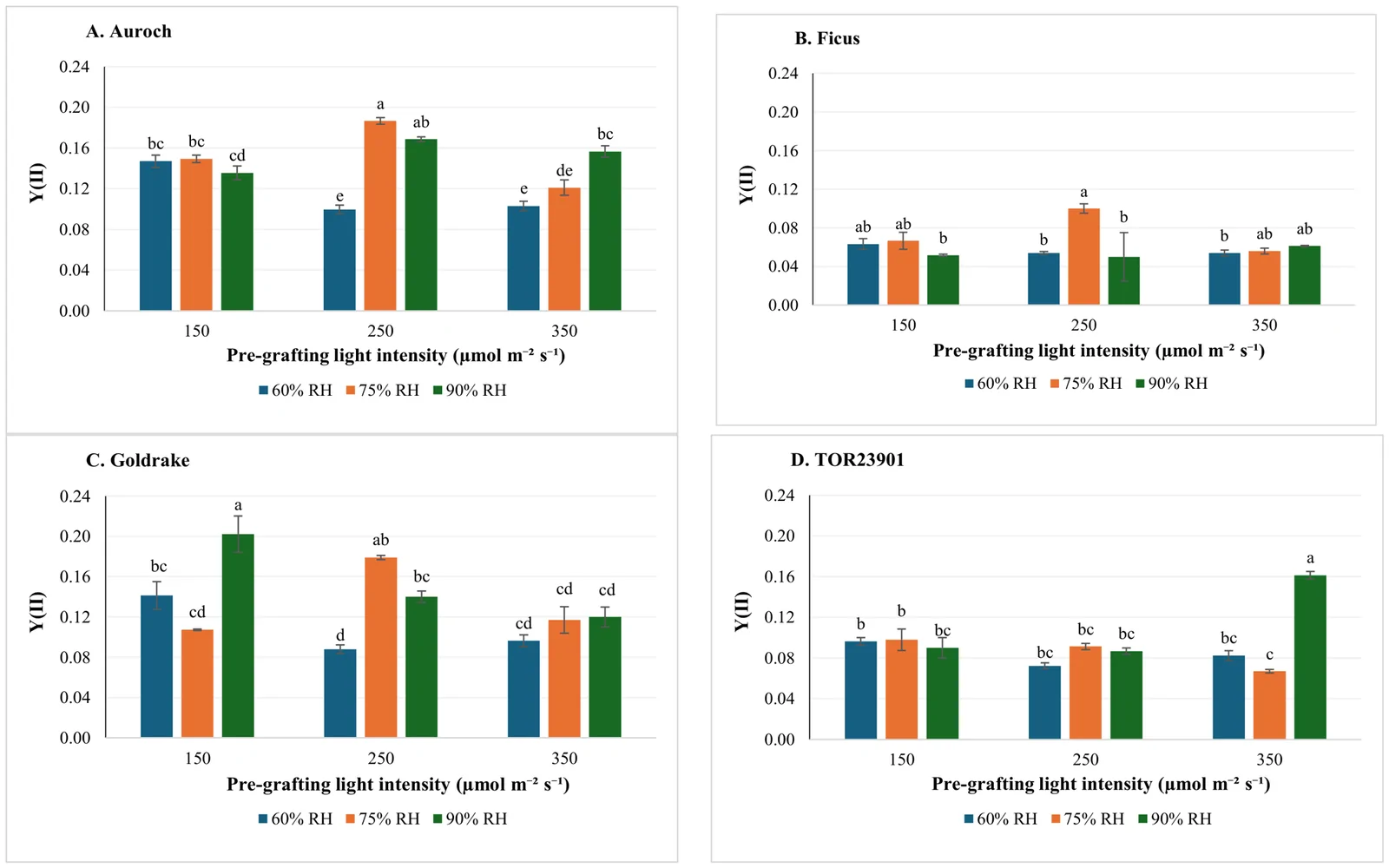
Effective quantum yield of PSII, Y(II), under pre-grafting light intensities and healing RH levels in (**A**) ‘Auroch’ (**B**) ‘Ficus’ (**C**) ‘Goldrake’ and (**D**) ‘TOR23901’. Values were obtained at an actinic irradiance of 335 µmol m^−2^·s^−1^ and are presented as the mean ± SE (*n* = 3). Different lowercase letters indicate significant differences among light × RH combinations according to Tukey’s HSD test (*p* < 0.05).

**Figure 5:**
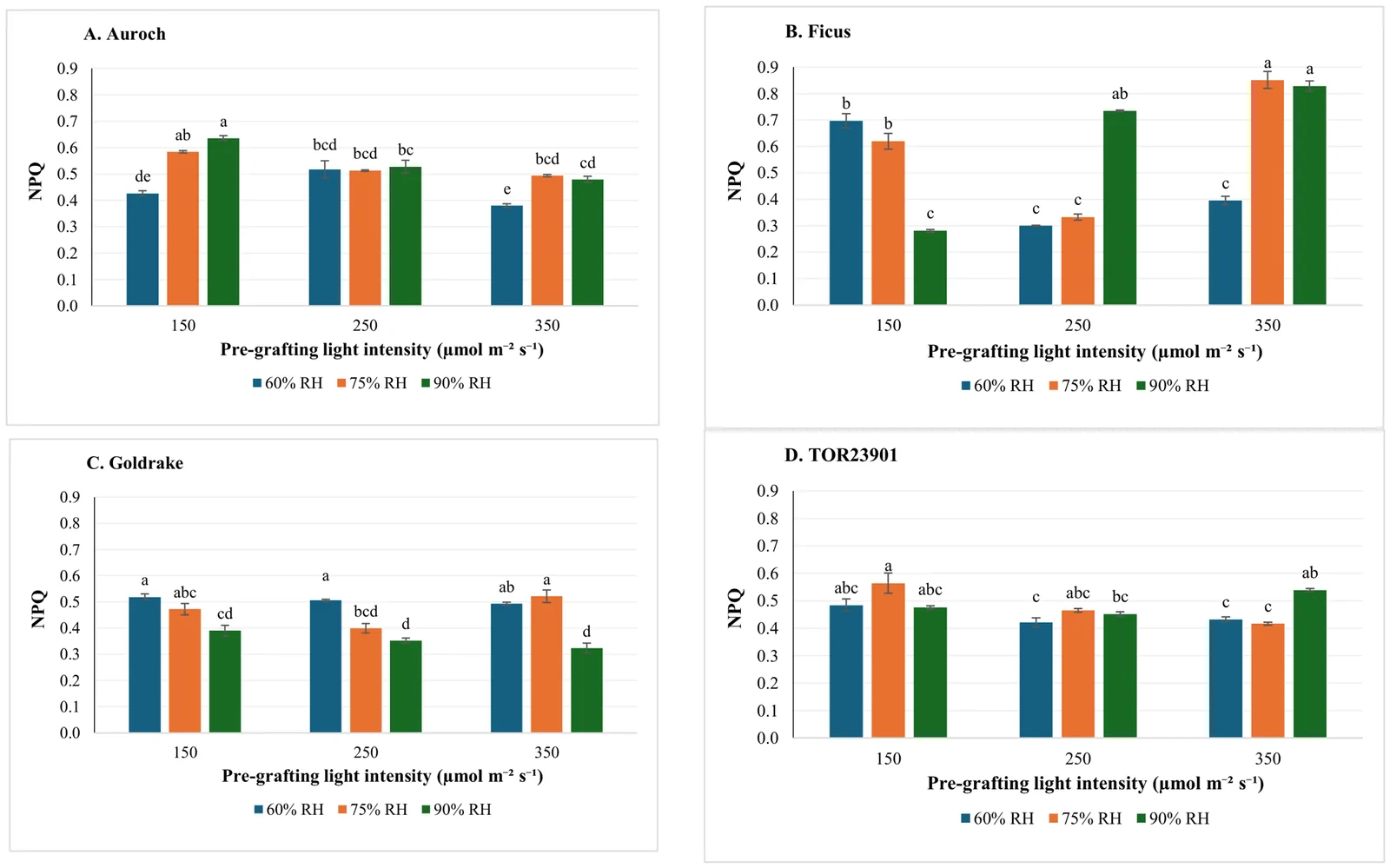
Non-photochemical quenching, NPQ, under pre-grafting light intensities and healing RH levels in (**A**) ‘Auroch’ (**B**) ‘Ficus’ (**C**) ‘Goldrake’ and (**D**) ‘TOR23901’. Values were obtained at an actinic irradiance of 335 µmol m^−2^·s^−1^ and are presented as the mean ± SE (*n* = 3). Different lowercase letters indicate significant differences among light × RH combinations according to Tukey’s HSD test (*p* < 0.05).

**Figure 6:**
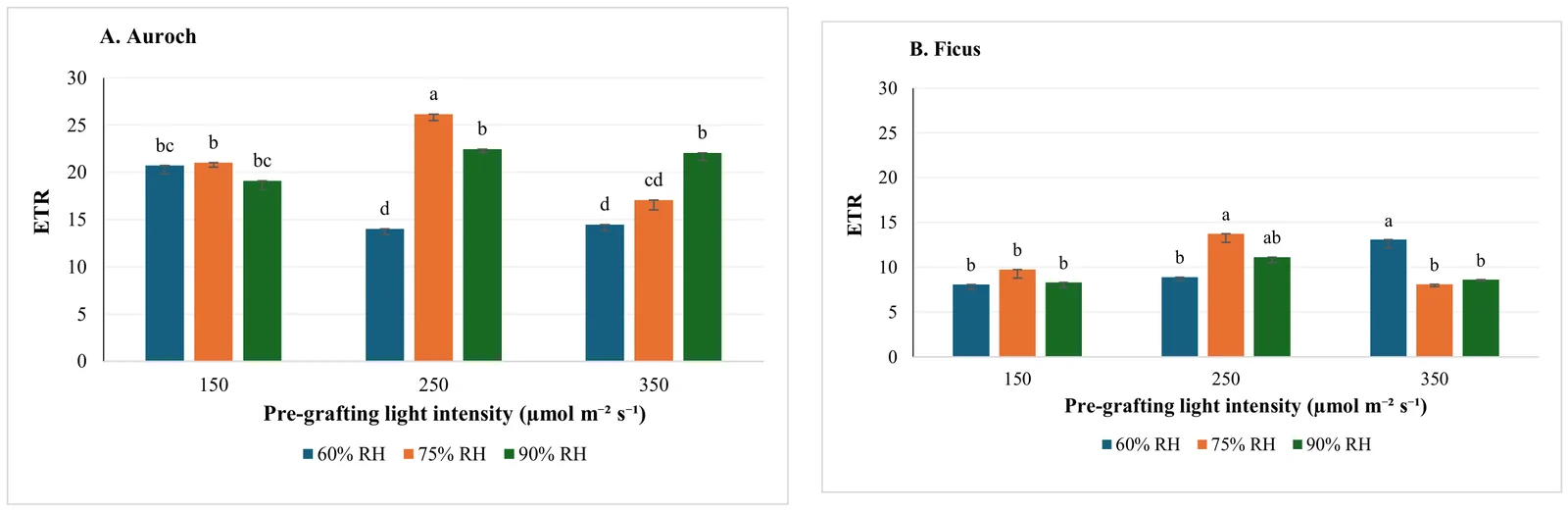

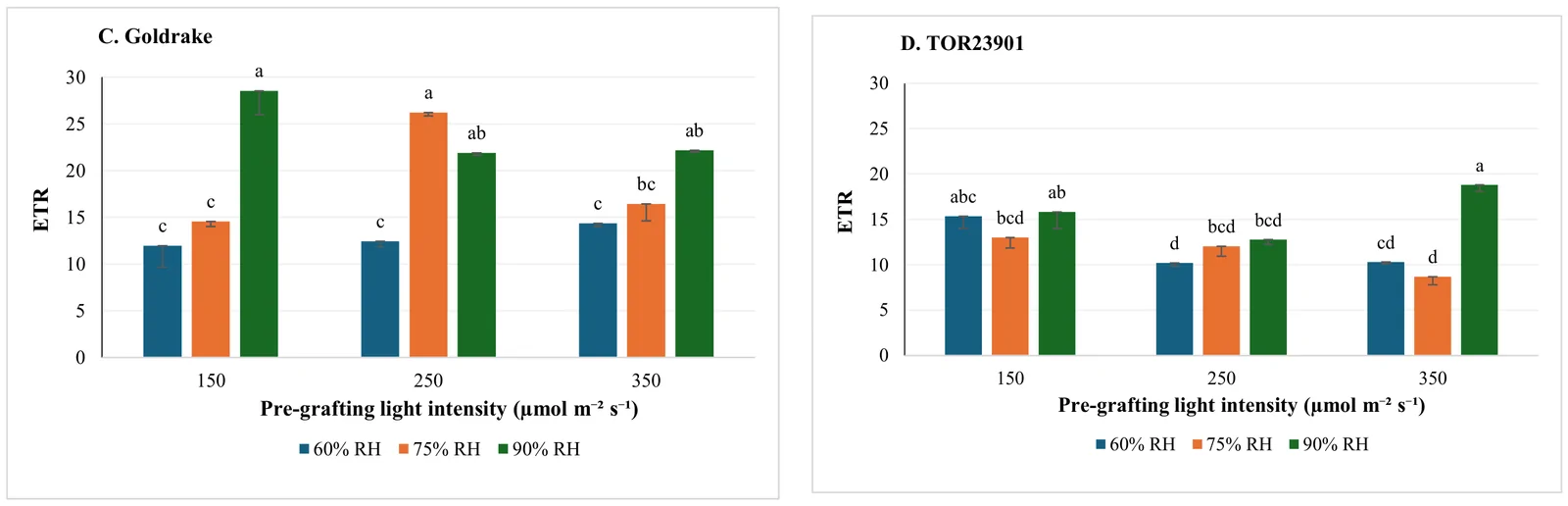
Electron transport rate, ETR, under pre-grafting light intensities and healing RH levels in (**A**) ‘Auroch’ (**B**) ‘Ficus’ (**C**) ‘Goldrake’ and (**D**) ‘TOR23901’. Values were obtained at an actinic irradiance of 335 µmol m^−2^·s^−1^ and are presented as the mean ± SE (*n* = 3). Different lowercase letters indicate significant differences among light × RH combinations according to Tukey’s HSD test (*p* < 0.05).

Chlorophyll fluorescence measured at a common actinic irradiance of 335 µmol m^−2^·s^−1^ showed that rootstock, pre-grafting light intensity, healing relative humidity, and their interactions significantly affected Y(II), NPQ, and ETR (Table 5). In particular, the significant rootstock × light intensity × relative humidity interaction for all three parameters indicated that the effects of pre-grafting light and healing humidity depended on rootstock (Figure 4, Figure 5 and Figure 6).

For the effective quantum yield of PSII [Y(II)], clear differences among light × RH combinations were observed within the individual rootstocks (Figure 2). In ‘Auroch’, maximum Y(II) was recorded in seedlings preconditioned at 250 µmol m^−2^·s^−1^ and subsequently healed at 75% RH (0.187). At the same pre-grafting light intensity, increasing healing relative humidity to 90% maintained a comparatively high Y(II) (0.163), whereas decreasing relative humidity to 60% reduced Y(II) to 0.10. At 350 µmol m^−2^·s^−1^, Y(II) increased with RH, reaching approximately 0.16 at 90% RH. At the lowest pre-grafting light intensity, differences among the three RH levels were comparatively smaller. In ‘Ficus’, Y(II) varied only slightly among the different light × RH combinations, with values ranging from 0.05 to 0.10. The highest value was recorded at 250 µmol m^−2^·s^−1^ + 75% RH (0.10), which was significantly higher than several treatments. ‘Goldrake’ exhibited a different response, with the highest Y(II) at 150 µmol m^−2^·s^−1^ + 90% RH (0.20), followed by 250 µmol m^−2^·s^−1^ + 75% RH (0.18). In contrast, the lowest value was noticed at 250 µmol m^−2^·s^−1^ + 60% RH (0.09). In ‘TOR23901’, Y(II) was greatest at 350 µmol m^−2^·s^−1^ with 90% RH (0.16), whereas the lowest response was observed at 350 µmol m^−2^·s^−1^ + 75% RH (0.07).

The non-photochemical quenching index (NPQ) was also strongly dependent on the interaction between pre-grafting light intensity and healing RH, and the response differed among rootstocks (Figure 3). In ‘Auroch’, the highest NPQ was observed under 150 µmol m^−2^·s^−1^ with 90% RH (0.64), followed by 150 µmol m^−2^·s^−1^ with 75% RH (0.58), whereas the lowest value was recorded at 350 µmol m^−2^·s^−1^ with 60% RH (0.38). At 250 µmol m^−2^·s^−1^, NPQ remained stable among the three relative humidity treatments (0.51–0.53). In ‘Ficus’, NPQ was significantly greater at 350 µmol photons m^−2^·s^−1^ combined with 75 and 90% RH, reaching 0.85 and 0.83, respectively. A relatively high value was also recorded at 250 µmol photons m^−2^·s^−1^ + 90% RH (0.73), whereas lower NPQ values occurred under 150 + 90%, 250 + 60%, and 250 + 75% RH.

In ‘Goldrake’, greater NPQ values were recorded at 150 and 250 µmol m^−2^·s^−1^ in combination with 60% RH, and at 350 µmol m^−2^·s^−1^ + 75% RH, with values ranging from 0.51 to 0.52. In contrast, NPQ decreased when 90% RH was combined with 250 or 350 µmol m^−2^·s^−1^, reaching 0.35 and 0.32, respectively. In TOR23901, NPQ reached a maximum of 0.56 at 150 µmol m^−2^·s^−1^ + 75% RH, while a comparable value was recorded at 350 µmol m^−2^·s^−1^ + 90% RH (0.54). Lower NPQ values, ranging from 0.42 to 0.43, were observed under 250 + 60%, 350 + 60%, and 350 + 75% RH.

The electron transport rate (ETR) showed a similar pattern observed for Y(II) (Figure 4). In ‘Auroch’, the greatest ETR was recorded at 250 µmol m^−2^·s^−1^ + 75% RH (26.13 µmol electrons m^−2^·s^−1^), which was significantly higher than all other treatment combinations. At the same pre-grafting light intensity, ETR decreased to 22.43 µmol electrons m^−2^·s^−1^ at 90% RH and to 14.00 µmol electrons m^−2^·s^−1^ at 60% RH. At 350 µmol m^−2^·s^−1^, ETR increased with increasing RH, reaching 22.03 µmol electrons m^−2^·s^−1^ at 90% RH. ‘Ficus’ exhibited lower ETR values than ‘Auroch’ and ‘Goldrake’. In ‘Ficus’, the greatest ETR values were recorded at 250 µmol m^−2^·s^−1^ + 75% RH (13.73 µmol electrons m^−2^·s^−1^) and 350 µmol m^−2^·s^−1^ + 60% RH (13.07) than other treatment combinations. ETR at 250 + 90% RH showed an intermediate response (11.13), whereas other treatment combinations showed lower ETR values.

‘Goldrake’ showed a different response pattern. The greatest ETR values were recorded at 150 µmol m^−2^·s^−1^ + 90% RH (28.53 µmol electrons m^−2^·s^−1^) and 250 µmol m^−2^·s^−1^ + 75% RH (26.20), both belonging to the same statistical group. ETR also remained relatively high at 250 + 90% RH (21.87) and 350 + 90% RH (22.17), with no significant difference from the two greatest values. In contrast, significantly lower ETR values were observed at 150 + 60%, 150 + 75%, 250 + 60%, and 350 + 60% RH, while 350 + 75% RH showed an intermediate response. In ‘TOR23901’, the maximum ETR was recorded at 350 µmol m^−2^·s^−1^ + 90% RH (18.80 µmol electrons m^−2^·s^−1^) and was significantly higher than several other light × RH combinations. In contrast, the lowest ETR values were observed at 250 + 60% RH and 350 + 75% RH, reaching 10.20 and 8.67 µmol electrons m^−2^·s^−1^, respectively.

Overall, the fluorescence responses differed markedly among the four rootstocks. The 250 µmol m^−2^·s^−1^ + 75% RH treatment produced the greatest Y(II) and ETR in ‘Auroch’ and a favorable response in ‘Ficus’ and ‘Goldrake’, whereas ‘TOR23901’ showed its greatest Y(II) and ETR at 350 µmol photons m^−2^·s^−1^ + 90% RH. NPQ showed a different pattern among rootstocks, further supporting the significant rootstock × light intensity × relative humidity interaction.

### 2.5. Correlation Analysis

**Figure 7:**
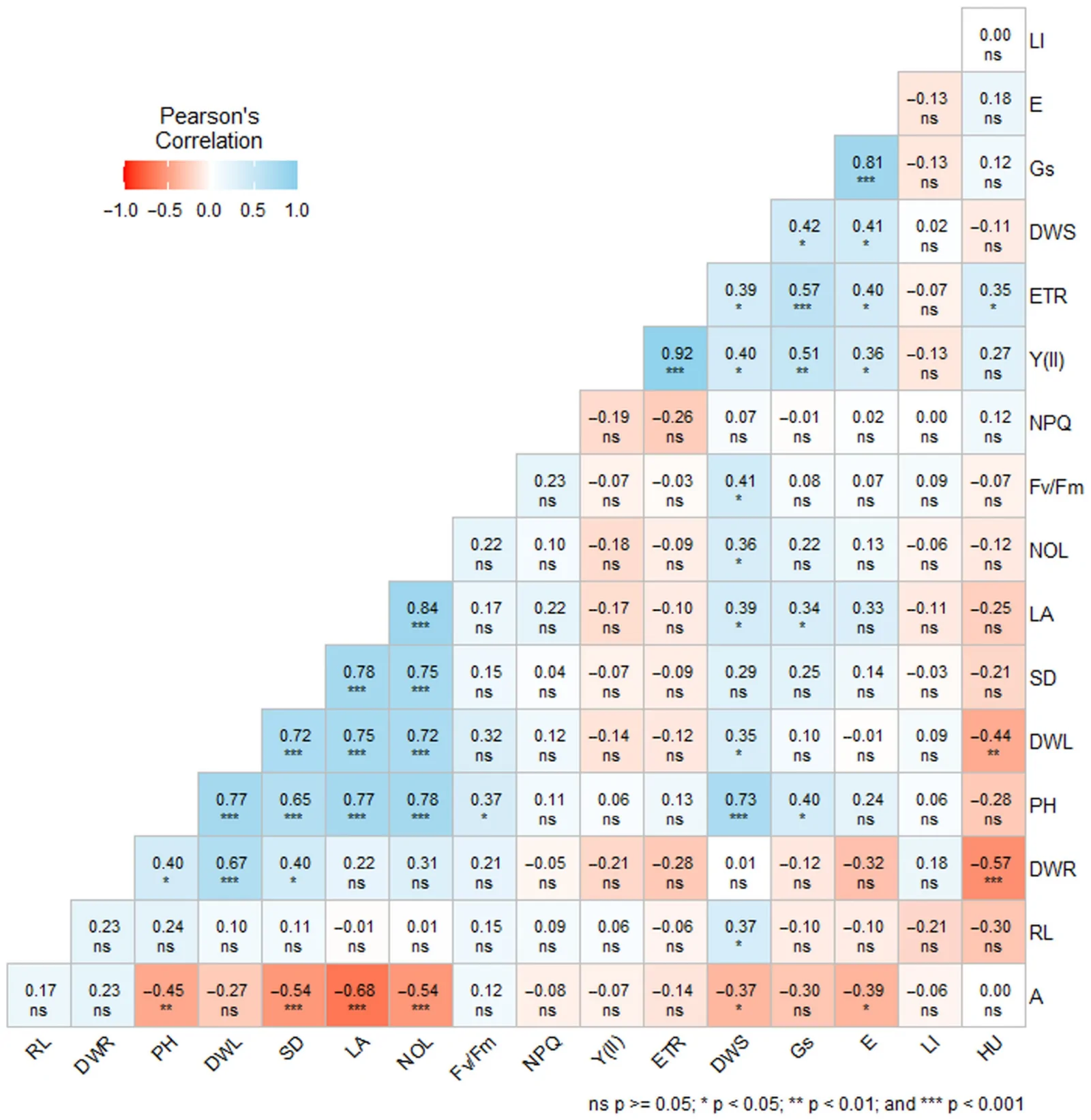
Pearson’s correlation matrix among growth, biomass, gas-exchange and chlorophyll fluorescence traits of grafted tomato seedlings. Correlation coefficients were calculated from treatment means (*n* = 36; 4 rootstocks × 3 pre-grafting light intensities × 3 healing relative humidities). Pre-grafting light intensity (LI; 150, 250 and 350 µmol m^−2^·s^−1^) and healing relative humidity (HU; 60, 75 and 90%) were included as numeric variables. Blue and red indicate positive and negative correlations, respectively. * *p* < 0.05; ** *p* < 0.01; *** *p* < 0.001; ns, not significant (*p* ≥ 0.05). PH, plant height; RL, root length; LA, leaf area; SD, stem diameter; NOL, number of leaves; DWR, root dry weight; DWS, stem dry weight; DWL, leaf dry weight; A, net photosynthetic rate; Gs, stomatal conductance; E, transpiration rate; Fv/Fm, maximum quantum efficiency of photosystem II; Y(II), effective quantum yield of photosystem II; NPQ, non-photochemical quenching; ETR, electron transport rate.

Pearson’s correlation analysis revealed strong positive relationships among several growth and physiological traits (Figure 7). Plant height was positively correlated with leaf dry weight, stem diameter, leaf area, number of leaves, and stem dry weight. Leaf area was also strongly associated with stem diameter and number of leaves, indicating coordinated vegetative development. Among the photosynthetic traits, Y(II) showed a very strong positive correlation with ETR. ETR was also positively correlated with stomatal conductance, transpiration rate and stem dry weight. Stomatal conductance and transpiration rate were strongly positively correlated. In contrast, photosynthetic rate (A) was negatively correlated with plant height, stem diameter, leaf area, number of leaves and stem dry weight. Healing relative humidity showed a significant negative correlation with root dry weight and leaf dry weight, whereas its association with ETR was positive.

## 3. Discussion

The present study demonstrated that graft success and post-grafting physiological performance of tomato seedlings were influenced by the combined effect of rootstock, pre-grafting light intensity, and healing relative humidity. These findings indicate that grafted seedling performance is determined not only by rootstock–scion compatibility, but also by the physiological condition of seedlings before grafting and the environmental conditions provided during healing. Seedlings grown under 250–350 µmol m^−2^·s^−1^ before grafting and healed at 75% relative humidity resulted in more uniform graft success across treatments, improved growth and photosynthetic performance (Figure 1). Similar responses have been reported in grafted tomato seedlings, where controlled recovery environments improved shoot dry weight, photosynthetic rate, water-use efficiency, and chlorophyll fluorescence responses [21]. Therefore, both pre-grafting seedling conditioning and post-grafting humidity management are important for producing vigorous grafted tomato seedlings.

Healing relative humidity affected graft establishment because water transport between the scion and rootstock is temporarily interrupted immediately after grafting. High RH is usually required during the early healing phase to reduce scion water loss and maintain tissue hydration until callus formation and vascular reconnection occur [21]. However, the present results showed that the highest humidity level was not always the most favorable. Graft success was most consistent at 250 µmol m^−2^·s^−1^ combined with 75% RH and at 350 µmol m^−2^·s^−1^ combined with 60–75% RH, whereas 90% RH produced more variable responses, especially in ‘Ficus’. (Table 1 and Table 3). This suggests that high healing RH may limit acclimatization and may not be equally suitable for all rootstock cultivars. Prolonged exposure to very high air humidity during leaf development impairs stomatal responsiveness and closure capacity [25], which may reduce the ability of grafted seedlings to regulate water loss after healing. In contrast, low humidity (60%) increases scion water loss before hydraulic reconnection is restored. Similarly, Vu et al. [14] reported that 90% RH during only the first 2–3 days after grafting, followed by a gradual reduction to 70%, improved graft-take and seedling quality, and other studies indicate that RH above 85% is not necessary for successful tomato graft healing and that healing conditions should be optimized rather than maximized [13,26]. Thus, 75% RH provided a better balance between graft survival, growth, and photosynthetic recovery than 90% RH, particularly when combined with 250 µmol m^−2^·s^−1^ pre-grafting light intensity.

The response to pre-grafting light intensity suggests that seedlings entered the healing phase with different physiological quality. Here, physiological quality refers to the condition of seedlings at grafting and during healing, assessed by measurable traits: stem diameter and dry matter accumulation (reflecting assimilate reserves and mechanical support), leaf area, PSII photochemical efficiency (Fv/Fm) and stomatal function (gs). Seedlings grown at 250 µmol m^−2^·s^−1^ showed the greatest plant height, root length, stem diameter and stem dry weight, and the highest net photosynthetic rate on average, whereas 350 µmol m^−2^·s^−1^ reduced leaf area, root length and stomatal conductance (Table 1 and Table 3). Moderate light may provide sufficient assimilate reserves to support callus formation and vascular reconnection without imposing excessive physiological demand after grafting [27]. In contrast, seedlings acclimated to higher light may experience a greater imbalance between absorbed light energy and the temporarily restricted water supply after grafting, promoting stomatal closure and delaying photosynthetic recovery [28]. A study examining tomato rootstocks grown at 150, 250 and 350 µmol m^−2^·s^−1^ showed optimal growth and biomass at 250 µmol m^−2^·s^−1^, while 350 µmol m^−2^·s^−1^ improved some photosynthetic traits without consistently increasing growth [17]. Light intensity, photoperiod, light quality and daily light integral have also been shown to affect tomato seedling morphology, stem development, photosynthetic capacity and post-grafting growth [29,30,31]. Graft-union formation also requires the differentiation of new xylem across the graft interface, a process driven by polar auxin flow from young leaves [27,32]. Pre-grafting light intensity may therefore have influenced vascular reconnection indirectly through its effects on stem development and assimilate supply, as reflected by the greater stem diameter and stem dry weight at 250 µmol m^−2^·s^−1^.

Growth and dry matter accumulation also differed among rootstocks, showing that grafted seedling vigor was not only determined by graft success. Among rootstocks, ‘Auroch’ accumulated the highest stem dry matter, whereas ‘Ficus’ produced a larger leaf area, thicker stems and the highest leaf dry weight. These differences suggest that different rootstocks contributed to post-grafting growth through distinct physiological strategies, ‘Auroch’ mainly promoted biomass accumulation, while ‘Ficus’ favored leaf expansion and stem thickening. Such differences may reflect variation in rootstock vigor, root hydraulic conductivity, and sink–source relationships, which are known to influence the allocation of assimilates and subsequent shoot development in grafted plants. A recent study showed similar rootstock-dependent differences in plant height, leaf area, stem diameter, and biomass accumulation [17]. Research studies indicate that tomato seedling growth traits respond strongly to RH and radiation quality [33]. Therefore, the observed growth responses were likely the result of interactions between rootstock-specific vigor and the physiological conditioning imposed by pre-grafting light intensity and healing environment.

Gas-exchange responses further demonstrated that post-grafting recovery was associated with both pre-grafting seedling condition and healing humidity. The highest photosynthetic rate was observed at 250 µmol m^−2^·s^−1^ and 75% RH (Table 3), indicating that moderate light conditioning before grafting and moderate humidity during healing supported better physiological recovery. At 75% RH, higher stomatal conductance and transpiration rate also suggest improved stomatal function after healing. This may indicate a more rapid restoration of hydraulic connectivity between the scion and rootstock, allowing stomata to resume regulation of gas exchange and water loss more effectively after graft-union formation. Therefore, treatments that supported earlier recovery of gas-exchange parameters likely promoted faster functional reconnection of the grafted partners. A study reported that high relative humidity widened stomatal apertures under high light intensity during tomato graft healing without increasing the transpiration rate [34]. Another study demonstrated that artificial light improved net photosynthetic rate and water-use efficiency during grafted tomato recovery [21]. Research studies indicate that suitable DLI during tomato graft healing improved graft-union adhesion, stem flow, total chlorophyll content, net photosynthetic rate, and biomass accumulation [31]. Together, these findings suggest that successful post-grafting recovery depends not only on maintaining adequate hydration during healing but also on stem development, assimilate reserves and photosynthetic competence of seedlings before grafting. The superior performance observed under 250 µmol m^−2^·s^−1^ and 75% RH indicates that moderate pre-grafting light and intermediate humidity may provide a favorable balance between water conservation, stomatal recovery, and photosynthetic reactivation following graft-union formation.

Chlorophyll fluorescence responses provided additional evidence that grafted seedling recovery was strongly influenced by the interaction between rootstock genotype, pre-grafting light intensity, and healing relative humidity. The higher Fv/Fm values recorded at 75% RH suggest that this humidity level favored the recovery of PSII photochemical efficiency following grafting, whereas both lower (60%) and higher (90%) humidity conditions may have imposed mild physiological constraints on photosynthetic recovery. Similar rootstock-dependent differences in chlorophyll fluorescence parameters have previously been reported in tomato rootstocks exposed to different intensity levels, indicating that rootstocks differ in their capacity to maintain photochemical activity under varying environmental conditions [17].

The combined behavior of Y(II), NPQ, and ETR indicates that pre-grafting light intensity influenced not only seedling morphology before grafting but also the post-grafting balance between photochemical energy utilization and photoprotective energy dissipation. Treatments combining higher Y(II) and ETR with moderate NPQ generally showed superior growth and graft success, indicating more efficient utilization of absorbed light energy for photosynthesis rather than thermal dissipation. Conversely, treatments characterized by elevated NPQ coupled with lower Y(II) and ETR appeared to allocate a greater proportion of excitation energy to photoprotective mechanisms, suggesting less efficient photosynthetic recovery following grafting. Across treatments, Y(II) was low (mostly 0.05–0.20), whereas NPQ remained moderate (approximately 0.3–0.85). Because absorbed light energy is partitioned among photochemistry [Y(II)], regulated non-photochemical dissipation [Y(NPQ)] and non-regulated dissipation [Y(NO)] [35], this combination indicates that a large fraction of absorbed energy was dissipated through non-regulated pathways and that a high proportion of PSII reaction centers was closed. This appears to be a main characteristic of light energy utilization in the grafted seedlings, consistent with a limited capacity to use absorbed light energy and to activate regulated photoprotection while vascular and hydraulic connections between scion and rootstock were still being re-established.

In ‘Auroch’, the combination of 250 µmol m^−2^·s^−1^ and 75% RH resulted in the highest Y(II) and ETR values, indicating effective use of absorbed light energy for photochemistry. At 150 µmol m^−2^·s^−1^ combined with 90% RH, NPQ increased while Y(II) and ETR declined, suggesting that a greater proportion of excitation energy was dissipated through non-photochemical pathways. Similar effects of light environment on Y(II), ETR, and related fluorescence traits have been reported in grafted tomato seedlings [9,36].

The response of ‘Ficus’ differed from that of ‘Auroch’. Although NPQ increased under 350 µmol m^−2^·s^−1^ combined with 75% or 90% RH, Y(II) and ETR remained relatively low, indicating a greater allocation of excitation energy to thermal dissipation. However, elevated NPQ should not necessarily be interpreted as evidence of photoinhibition, as non-photochemical quenching represents an important photoprotective mechanism that prevents excess excitation pressure on PSII [9,37]. The highest Y(II) and ETR values in ‘Ficus’ were observed at 250 µmol m^−2^·s^−1^ and 75% RH, suggesting more efficient photochemical use of absorbed light under this treatment.

‘Goldrake’ and ‘TOR23901’ exhibited different patterns, further highlighting the importance of genotype-specific responses. In ‘Goldrake’, both 150 µmol m^−2^·s^−1^ + 90% RH and 250 µmol m^−2^·s^−1^ + 75% RH maintained high Y(II) and ETR, whereas increasing RH to 90% under higher pre-grafting PPFD reduced NPQ and increased ETR. However, ‘Goldrake’ showed very low Fv/Fm values at 150 µmol m^−2^·s^−1^ with 60% RH and at 350 µmol m^−2^·s^−1^ with 90% RH, well below the values typical of unstressed leaves [38], indicating chronic photoinhibition or severe physiological stress. This interpretation is supported by the reduced net photosynthetic rate and growth of ‘Goldrake’ under these conditions. The relatively high stomatal conductance at 350 µmol m^−2^·s^−1^ with 90% RH, despite low Fv/Fm and photosynthesis, suggests that photosynthesis was limited mainly by PSII damage rather than by stomatal closure. These two treatment combinations therefore appear to have imposed stress on the ‘Goldrake’ graft combination, leading to reduced PSII efficiency and growth. In contrast, ‘TOR23901’ achieved its highest Y(II) and ETR at 350 µmol m^−2^·s^−1^ combined with 90% RH, indicating that this rootstock maintained efficient PSII activity under stronger pre-grafting light conditions while simultaneously dissipating excess excitation energy through photoprotective mechanisms.

Collectively, these responses indicate that the physiological condition established before grafting strongly influenced the balance between photochemical energy utilization and photoprotective energy dissipation during the healing phase. The superior fluorescence performance observed under 250 µmol m^−2^·s^−1^ combined with 75% RH in several rootstocks suggests that moderate PPFD before grafting may enhance photosynthetic competence without inducing excessive photoprotective energy dissipation after healing. The negative correlations between net photosynthetic rate (A) and growth traits in the pooled analysis (Figure 7) were largely driven by differences among rootstocks, as ‘TOR23901’ combined the highest A with the smallest plant size and the lowest stomatal conductance and transpiration rate (Table 1 and Table 3). Rootstocks modify scion growth through differences in root vigor, water and nutrient uptake and hormonal signaling [39]. However, the comparatively high A observed in ‘TOR23901’ together with very low stomatal conductance appears physiologically unusual and is not fully consistent with the expected limitation of CO_2_ diffusion through stomata. Within individual rootstocks, A was positively associated with stomatal conductance and transpiration rate, particularly in ‘Auroch’ and ‘TOR23901’. Moreover, gas-exchange measurements represent instantaneous photosynthetic activity, whereas growth traits reflect cumulative carbon gain integrated over the entire cultivation period, so the two are often poorly correlated [40]. In addition, the correlations between A and the chlorophyll fluorescence parameters Y(II) and ETR were weak and non-significant, indicating that variation in PSII photochemical efficiency was not directly translated into CO_2_ assimilation. This may reflect the use of photosynthetic electrons in alternative sinks such as photorespiration and photoprotective processes, as well as differences between the measurement conditions of chlorophyll fluorescence imaging and leaf gas exchange [41].

Overall, the results indicate that successful graft recovery depends on the interaction between rootstock genotype, pre-grafting light environment, and healing relative humidity. Pre-grafting light intensity influenced stem development, dry matter accumulation and photosynthetic competence of seedlings entering the healing phase, whereas healing humidity determined the efficiency of graft-union establishment and post-grafting physiological recovery. Among the tested treatments, 250 µmol m^−2^·s^−1^ combined with 75% RH during healing provided the most consistent overall balance of high graft success, vigorous seedling growth, and favorable physiological performance across the four rootstocks. Although 350 µmol m^−2^·s^−1^ also supported high graft success under 60–75% RH, it did not provide comparable improvements across all measured traits. These findings suggest that moderate pre-grafting PPFD combined with intermediate healing humidity provides a favorable balance between hydration, acclimatization, and photosynthetic recovery, supporting the production of high-quality grafted tomato seedlings.

## 4. Materials and Methods

### 4.1. Plant Material and Growing Conditions

The experiment was conducted at the Institute of Horticulture, Lithuanian Research Centre for Agriculture and Forestry under controlled environment conditions. The tomato scion cultivar ‘Jalila F1’ (SAKATA, France, Uchaud) was grafted onto four tomato rootstocks: ‘Goldrake F1’, ‘TOR23901’ (ESASEM, Italy, Casaleone, Verona), ‘Auroch’ and ‘Ficus’ (SAKATA, France, Uchaud). Among the rootstocks used in the present study, ‘Goldrake’ is a well-established commercial rootstock with high disease resistance, whereas ‘Ficus’ is a vigorous rootstock commonly used for medium- and long-cycle cultivation because of its strong vegetative capacity and yield potential. ‘Auroch’ and ‘TOR23901’ are relatively newer rootstocks described as having high disease resistance and potential for maintaining growth during long production cycles. Seeds were sown in 0.5 L pots filled with peat substrate. The experiment was laid out in a completely randomized design (CRD) with three replicates per treatment combination, each containing five seedlings (15 seedlings per treatment combination). After the pre-grafting light treatments, nine seedlings per treatment combination (three per replicate) were grafted and transferred to the healing chambers, and graft success was calculated from these nine plants. One plant per replicate was used for the subsequent measurements, and the replicate was considered the experimental unit (*n* = 3). The average concentration of primary nutrients in the substrate was N, 110 mg L^−1^; P_2_O_5_, 50 mg L^−1^ and K_2_O, 160 mg L^−1^ with sufficient microelements Fe, Mn, Cu, B, Mo, and Zn. The electrical conductivity (EC) varied between 1.0 and 2.5 m S/cm (‘Durpeta’ peat substrate, Netoniai, Lithuania). Plants were watered as required to maintain uniform substrate moisture during the pre-grafting period.

### 4.2. Pre-Grafting Light Conditioning

The light treatments were applied during the pre-grafting seedling growth stage. Plants were grown in a controlled-environment walk-in chamber at +23/19 °C day/night temperature and 55–60% relative air humidity, with a 16 h thermoperiod and photoperiod. Pre-grafting PPFD treatments of 150, 250, and 350 µmol m^−2^·s^−1^ were provided using standard high-pressure sodium lamps (HPS) (SON-T Agro, 600 W, Philips, Eindhoven, The Netherlands). PPFD was measured and regulated at the plant level using a photometer–radiometer (RF-100, Sonopan, Bialystok, Poland) during rootstock growth. Seedlings were grafted approximately 18 days after sowing when plants had an average of 3–4 true leaves. Healthy and visually uniform rootstock and scion seedlings with comparable stem thickness at the grafting position, overall size, and developmental stage were selected for grafting.

### 4.3. Grafting Procedure and Healing Conditions

Grafting was performed using the splicing method. The rootstock seedlings were cut about 1 cm below the cotyledon leaves, and scion seedlings were cut about 0.5 cm above the cotyledonary leaves. The scions and rootstocks were cut at an angle of approximately 45 and grafting clips were used to adhere to the graft union [42]. After grafting, grafted seedlings from each pre-grafting light treatment were immediately transferred to healing chambers in which they were exposed to three relative humidity levels: 60 ± 5%, 75 ± 5% and 90 ± 5%. The three RH regimes were maintained in three separate healing chambers, with one chamber assigned to each RH level. Each chamber contained two shelves, and RH was monitored continuously using digital sensors and controlled using humidifiers. Grafted seedlings were arranged randomly within each chamber to minimize positional effects.

The healing chambers were constructed from transparent polycarbonate and had no supplemental lighting; thus, grafted seedlings were exposed to the ambient growth-chamber light (16 h photoperiod) transmitted through the chamber cover, which was identical for all treatments. PPFD inside the healing chambers was not recorded. The air temperature during healing was maintained at 23/19 °C day/night. After 7 days in the healing chamber, grafted seedlings were transferred back to the growth chamber for post-grafting growth. Post-grafting measurements were performed 15 days after healing. Pre-grafting PPFD was applied before grafting, while relative humidity was applied during the healing phase. All reported measurements were collected after grafting and acclimatization.

### 4.4. Graft Success and Biometric Measurements

Graft success was recorded after the healing and acclimatization period. Graft success percentage was calculated as:

(1) $$\mathrm{Graft}\;\mathrm{success}\;(\%)=\frac{\mathrm{Number}\;\mathrm{of}\;\mathrm{successfully}\;\mathrm{healed}\;\mathrm{grafted}\;\mathrm{plants}}{\mathrm{Total}\;\mathrm{number}\;\mathrm{of}\;\mathrm{grafted}\;\mathrm{plants}}\times 100$$

Graft success percentage was calculated for each treatment as the proportion of successfully healed grafted plants relative to the total number of grafted plants (*n* = 9 per treatment) and is presented in Figure 1.

Biometric measurements were performed on one plant per replicate, with three replicates per treatment (*n* = 3). Leaf area was measured using an automatic leaf area meter (Delta-T Devices, Wallingford, UK). Plant height was measured using a ruler with an accuracy of 1 mm. To measure the roots, the substrate was removed from the plant roots, they were carefully and thoroughly washed, and their length was measured using a ruler with an accuracy of 1 mm. The fresh weight (FW) of roots, stems, and leaves was determined immediately after sampling using an analytical balance with an accuracy of 0.001 g. The plant material was then lyophilized (FD-7, SIA Cryogenic and Vacuum Systems, Latvia), and the dry weight (DW) of roots, stems, and leaves was recorded after lyophilization. The diameter of the plant stem was measured with a caliper (Mitutoyo Europe GmbH in Neuss, Germany) (accuracy of 0.1 mm) above the first embryonic leaves.

### 4.5. Gas-Exchange Measurements

Leaf gas-exchange parameters photosynthetic rate (Pr, μmol CO_2_ m^−2^·s^−1^), transpiration rate (Tr, mmol·H_2_O·m^−2^·s^−1^), stomatal conductance (gs, mol·H_2_O·m^−2^·s^−1^), and internal CO2 concentration (Ci, ppm) were measured using a portable photosynthesis system (LI-COR 6400XT, Lincoln, NE, USA) equipped with a red/blue LED light source (6400-02B). Leaf chamber conditions were set to a block temperature of 23 °C, a reference CO_2_ concentration of 400 µmol mol^−1^, a relative humidity of approximately 60%, a flow rate of 500 µmol s^−1^ and a near-saturating PPFD of 1000 µmol m^−2^·s^−1^. Measurements were performed on the third fully expanded leaf between 9:00 and 12:00 a.m. One plant was measured per replicate, with three replicates per treatment. For each plant, measurements were recorded for 1 min (19 measurements per plant), and these readings were averaged to obtain one value per plant. These values (*n* = 3) were used for statistical analysis.

### 4.6. Chlorophyll Fluorescence Measurements

Chlorophyll fluorescence was measured using an imaging-PAM fluorometer (M-Series MAXI-Version; Walz, Effeltrich, Germany) [43,44]. Before measurements, plants were dark-adapted for 30–40 min to minimize all quenching processes. Rapid light curves (RLCs) were recorded over actinic irradiances ranging from 0 to 1251 µmol photons m^−2^·s^−1^. Fluorescence parameters were recorded at 20 s intervals (clock time 20 s) during a 315 s measurement sequence. The actinic light intensity setting was 8, while saturating pulses were applied at intensity setting 7 with a pulse duration of 240 ms (4 × 60 ms). The effective quantum yield of PSII [*Y*(*II*)], non-photochemical quenching (NPQ) and electron transport rate (ETR) were calculated automatically using ImagingWin software V2.56zm based on steady-state fluorescence (F) and maximum fluorescence under light-adapted conditions (*F_m_*′). The values presented here correspond to the 335 µmol photons m^−2^·s^−1^ step, which was selected for comparison among treatments. Measurements were taken on one plant per replicate, with three replicates per treatment; these values (*n* = 3) were used for statistical analysis. The minimum fluorescence (*F*_0_) and maximum fluorescence (*F_m_*) were recorded in dark-adapted leaves, and the maximum quantum efficiency of photosystem II was calculated as:

(2) $$F_{v}/F_{m}=\frac{F_{m}-F_{0}}{F_{m}}$$

Light-adapted fluorescence parameters were recorded under actinic light. The effective quantum yield of PSII, *Y*(*II*), was calculated as:

(3) $$Y(\mathrm{II})=\frac{F_{m}^{\prime }-F^{\prime }}{F_{m}^{\prime }}$$

where:

- *F*^′^ = steady-state fluorescence yield under actinic light.
- $F_{m}^{\prime }$ = maximum fluorescence yield obtained during a saturating pulse under the same light.

Non-photochemical quenching (*NPQ*) was calculated as Murchie et al. [44].

(4) $$\mathrm{NPQ}=\frac{F_{m}-F_{m}^{\prime }}{F_{m}^{\prime }}$$

where:

- *F_m_* = maximum fluorescence yield in the dark-adapted state.
- $F_{m}^{\prime }$= maximum fluorescence yield under actinic illumination.

Electron transport rate (ETR), was calculated as:

ETR = PAR × Y(II) × 0.5 × 0.84(5)

where:

- PAR = photosynthetically active radiation (μmol photons m^−2^·s^−1^).
- 0.5 assumes equal energy distribution between PSII and PSI.
- 0.84 represents the leaf absorptivity value.

### 4.7. Statistical Analysis

Data were analyzed using Microsoft Excel, Addinsoft XLSTAT 2025, and R statistical software version 4.5.2 (R Foundation for Statistical Computing, Vienna, Austria). The effects of rootstock, pre-grafting light intensity, healing relative humidity, and their interactions were evaluated using three-way analysis of variance (ANOVA). Differences among treatment means were assessed using Tukey’s honestly significant difference (HSD) test at *p* < 0.05. Pearson’s correlation analysis was used to assess relationships among growth, biomass, gas-exchange, and chlorophyll fluorescence traits.

## 5. Conclusions

Overall, the results indicate that successful graft recovery depends on the interaction between rootstock genotype, pre-grafting light intensity, and healing relative humidity. Among the tested treatments, 250 µmol m^−2^·s^−1^ combined with 75% RH during healing provided the most consistent overall balance of high graft success, vigorous seedling growth, and favorable physiological performance across the four rootstocks. However, responses differed among rootstocks, and some physiological traits were more favorable under other light × RH combinations. Although 350 µmol m^−2^·s^−1^ also supported high graft success under 60–75% RH, it did not consistently improve all measured traits. Therefore, balancing pre-grafting seedling conditioning with appropriate humidity management appears to be an effective strategy for producing high-quality grafted tomato seedlings.

## Figures and Tables

**Table 1:** Effects of rootstock, pre-grafting light intensity and healing relative humidity on growth of grafted tomato seedlings.

Factor	Level	Plant Height (cm)	Root Length (cm)	Leaf Area (cm^2^)	Stem Diameter (mm)	No. of True Leaves	Root Dry Weight (g)	Stem Dry Weight (g)	Leaf Dry Weight (g)
Rootstock	Auroch	22.11 ± 0.65 A	27.61 ± 0.89 A	207.12 ± 9.95 B	4.92 ± 0.10 C	5 ± 0.13 B	0.086 ± 0.009 B	1.049 ± 0.022 A	0.988 ± 0.063 AB
	Ficus	20.26 ± 0.46 B	24.19 ± 0.70 B	254.06 ± 6.03 A	5.41 ± 0.11 A	6 ± 0.08 A	0.105 ± 0.006 A	0.366 ± 0.016 B	1.081 ± 0.043 A
	Goldrake	18.13 ± 0.66 C	23.80 ± 0.70 B	191.97 ± 9.69 C	5.15 ± 0.17 B	5 ± 0.11 B	0.100 ± 0.010 AB	0.311 ± 0.024 C	0.974 ± 0.061 B
	TOR23901	15.30 ± 0.37 D	26.17 ± 0.65 A	100.59 ± 4.49 D	3.96 ± 0.07 D	5 ± 0.07 C	0.107 ± 0.005 A	0.206 ± 0.010 D	0.780 ± 0.028 C
Light intensity (µmol m^−2^·s^−1^)	150	18.31 ± 0.56 B	25.42 ± 0.59 B	196.74 ± 11.63 A	4.82 ± 0.13 AB	5 ± 0.10 A	0.091 ± 0.006 B	0.464 ± 0.058 B	0.919 ± 0.048 A
	250	19.69 ± 0.64 A	27.22 ± 0.77 A	190.30 ± 11.99 A	4.99 ± 0.15 A	5 ± 0.10 A	0.099 ± 0.008 AB	0.506 ± 0.054 A	0.976 ± 0.046 A
	350	18.85 ± 0.69 B	23.68 ± 0.55 C	178.27 ± 10.97 B	4.76 ± 0.12 B	5 ± 0.13 A	0.107 ± 0.007 A	0.479 ± 0.063 B	0.973 ± 0.048 A
Relative humidity (%)	60	19.33 ± 0.47 B	26.79 ± 0.73 A	201.57 ± 10.79 A	4.88 ± 0.11 B	5 ± 0.12 AB	0.116 ± 0.006 A	0.498 ± 0.053 B	1.027 ± 0.034 A
	75	20.65 ± 0.59 A	25.24 ± 0.60 B	202.71 ± 10.81 A	5.20 ± 0.14 A	6 ± 0.09 A	0.118 ± 0.005 A	0.543 ± 0.061 A	1.090 ± 0.043 A
	90	16.86 ± 0.66 C	24.29 ± 0.67 B	161.02 ± 11.78 B	4.49 ± 0.14 C	5 ± 0.12 B	0.065 ± 0.006 B	0.408 ± 0.059 C	0.752 ± 0.043 B

Values are means ± SE (*n* = 27 for rootstock; *n* = 36 for light intensity and relative humidity; each observation represents one plant per replicate). Within each factor, means in a column followed by different uppercase letters differ significantly according to Tukey’s HSD test (*p* < 0.05). Because the rootstock × light intensity × relative humidity interaction was significant for all traits (Table 2), treatment combinations within each rootstock are compared in Figure 2 and Table S3.

**Table 2:** Analysis of variance (F-values and *p* values) for growth and biomass traits of grafted tomato seedlings.

Source of Variation	df	Plant Height (cm)	Root Length (cm)	Leaf Area (cm^2^)	Stem Diameter (mm)	No. of True Leaves	Root Dry Weight (g)	Stem Dry Weight (g)	Leaf Dry Weight (g)
Rootstock (R)	3	176.43 (<0.001)	11.55 (<0.001)	257.21 (<0.001)	109.13 (<0.001)	38.90 (<0.001)	4.09 (0.010)	1787.23 (<0.001)	23.44 (<0.001)
Light intensity (LI)	2	13.44 (<0.001)	15.22 (<0.001)	7.30 (0.001)	5.00 (0.009)	0.56 (0.572)	4.17 (0.019)	7.59 (0.001)	2.02 (0.140)
Relative humidity (RH)	2	101.62 (<0.001)	7.73 (<0.001)	46.85 (<0.001)	47.31 (<0.001)	6.75 (0.002)	57.35 (<0.001)	75.88 (<0.001)	63.26 (<0.001)
R × LI	6	16.27 (<0.001)	4.61 (<0.001)	13.09 (<0.001)	7.33 (<0.001)	8.15 (<0.001)	3.38 (0.005)	7.58 (<0.001)	6.91 (<0.001)
R × RH	6	8.13 (<0.001)	3.48 (0.004)	9.96 (<0.001)	16.11 (<0.001)	4.58 (<0.001)	6.06 (<0.001)	10.37 (<0.001)	9.77 (<0.001)
LI × RH	4	3.86 (0.007)	2.27 (0.070)	4.37 (0.003)	4.93 (0.001)	0.84 (0.502)	5.27 (<0.001)	3.55 (0.011)	1.68 (0.165)
R × LI × RH	12	15.04 (<0.001)	2.70 (0.005)	5.66 (<0.001)	4.34 (<0.001)	2.80 (0.003)	3.60 (<0.001)	7.37 (<0.001)	6.84 (<0.001)
Error	72	–	–	–	–	–	–	–	–

Values are F-ratios with exact *p* values in parentheses from a three-way ANOVA with rootstock (R), pre-grafting light intensity (LI) and healing relative humidity (RH) as fixed factors (*n* = 3 replicates per treatment combination). df, degrees of freedom. Complete ANOVA results (SS, MS, F, P) are given in Table S1.

**Table 5:** Three-way ANOVA of the effects of rootstock, pre-grafting light intensity and healing relative humidity on PAM fluorescence parameters.

Source of Variation	df	Y(II)	NPQ	ETR
Rootstock (R)	3	194.127 (<0.0001)	53.609 (<0.0001)	199.059 (<0.0001)
Light intensity (LI)	2	8.768 (<0.0001)	25.739 (<0.0001)	7.697 (0.001)
Relative humidity (RH)	2	38.515 (<0.0001)	22.110 (<0.0001)	73.765 (<0.0001)
R × LI	6	8.132 (<0.0001)	34.290 (<0.0001)	6.371 (<0.0001)
R × RH	6	9.063 (<0.0001)	31.659 (<0.0001)	24.695 (<0.0001)
LI × RH	4	29.295 (<0.0001)	44.207 (<0.0001)	26.716 (<0.0001)
R × LI × RH	12	9.391 (<0.0001)	50.352 (<0.0001)	9.511 (<0.0001)
Error	72	–	–	–

Values are F-ratios with *p* values in parentheses from a three-way ANOVA with rootstock (R), pre-grafting light intensity (LI) and healing relative humidity (RH) as fixed factors. LI, pre-grafting light intensity; RH, healing relative humidity. Effects were considered significant at *p* < 0.05.

## Data Availability

The original contributions presented in this study are included in the article/Supplementary Material. Further inquiries can be directed to the corresponding author.

## Supplementary Materials

The following supporting information can be downloaded at: https://www.mdpi.com/article/10.3390/plants15213300/s1, Table S1: Complete analysis of variance for growth and biomass traits of grafted tomato seedlings; Table S2: Complete analysis of variance for gas exchange and chlorophyll fluorescence parameters of grafted tomato seedlings; Table S3: Growth and biomass traits of Jalila F1 tomato seedlings grafted onto four rootstocks under nine combinations of pre-grafting light intensity and healing relative humidity (RH); Table S4: Gas exchange and chlorophyll fluorescence parameters of Jalila F1 tomato seedlings grafted onto four rootstocks under nine combinations of pre-grafting light intensity and healing relative humidity (RH).

## Abbreviations

The following abbreviations are used in this manuscript:

A	Photosynthetic rate
Gs	Stomatal conductance
E	Transpiration rate
Fv/Fm	Maximum quantum efficiency of PSII
Y(II)	Effective quantum yield of PSII
NPQ	Non-photochemical quenching
ETR	Electron transport rate
LED	Light-emitting diode
PSII	Photosystem II
PPFD	Photosynthetic photon flux density
