# Supplementary material for: Effects of Rootstock, Pre-Grafting Light Intensity and Healing Relative Humidity on Photosynthetic Performance of Grafted Tomato Seedlings
Source: Plants (Basel). 2026 Oct 26;15(21):3300. doi: 10.3390/plants15213300 (PMC13765880; doi:10.3390/plants15213300)
Supplement: Supplementary file 1 [file plants-15-03300-s001.zip › plants-4611460-supplementary.pdf]

Table S1. Complete analysis of variance for growth and biomass traits of grafted tomato seedlings.

Trait	Source of variation	df	SS	MS	F	P
	Rootstock (R)	3	694.69	231.56	176.43	<0.001
	Light intensity (LI)	2	35.28	17.64	13.44	<0.001
	Relative humidity (RH)	2	266.75	133.38	101.62	<0.001
	R × LI	6	128.16	21.36	16.27	<0.001
**Plant height (cm)**	R × RH	6	64.02	10.67	8.13	<0.001
	LI × RH	4	20.25	5.06	3.86	0.007
	R × LI × RH	12	236.81	19.73	15.04	<0.001
	Error	72	94.50	1.31	–	–
	Total	107	1540.47	–	–	–
	Rootstock (R)	3	256.99	85.66	11.55	<0.001
	Light intensity (LI)	2	225.81	112.91	15.22	<0.001
	Relative humidity (RH)	2	114.74	57.37	7.73	<0.001
	R × LI	6	205.12	34.19	4.61	<0.001
**Root length (cm)**	R × RH	6	155.07	25.85	3.48	0.004
	LI × RH	4	67.33	16.83	2.27	0.070
	R × LI × RH	12	240.63	20.05	2.70	0.005
	Error	72	534.17	7.42	–	–
	Total	107	1799.86	–	–	–
	Rootstock (R)	3	334397.36	111465.79	257.21	<0.001
	Light intensity (LI)	2	6329.31	3164.65	7.30	0.001
	Relative humidity (RH)	2	40608.38	20304.19	46.85	<0.001
	R × LI	6	34033.81	5672.30	13.09	<0.001
**Leaf area (cm^2)**	R × RH	6	25906.01	4317.67	9.96	<0.001
	LI × RH	4	7571.58	1892.90	4.37	0.003
	R × LI × RH	12	29449.72	2454.14	5.66	<0.001
	Error	72	31202.71	433.37	–	–
	Total	107	509498.89	–	–	–
	Rootstock (R)	3	32.325	10.775	109.13	<0.001
	Light intensity (LI)	2	0.988	0.494	5.00	0.009
	Relative humidity (RH)	2	9.343	4.672	47.31	<0.001
	R × LI	6	4.344	0.724	7.33	<0.001
**Stem diameter (mm)**	R × RH	6	9.546	1.591	16.11	<0.001
	LI × RH	4	1.946	0.487	4.93	0.001
	R × LI × RH	12	5.144	0.429	4.34	<0.001
	Error	72	7.109	0.099	–	–
	Total	107	70.745	–	–	–
	Rootstock (R)	3	17.29	5.76	38.90	<0.001
	Light intensity (LI)	2	0.17	0.08	0.56	0.572
	Relative humidity (RH)	2	2.00	1.00	6.75	0.002
	R × LI	6	7.24	1.21	8.15	<0.001
**No. of true leaves**	R × RH	6	4.07	0.68	4.58	<0.001
	LI × RH	4	0.50	0.12	0.84	0.502
	R × LI × RH	12	4.98	0.42	2.80	0.003
	Error	72	10.67	0.15	–	–
	Total	107	46.92	–	–	–
	Rootstock (R)	3	0.00681	0.00227	4.09	0.010
	Light intensity (LI)	2	0.00462	0.00231	4.17	0.019
**Root dry weight (g)**	Relative humidity (RH)	2	0.06364	0.03182	57.35	<0.001
	R × LI	6	0.01124	0.00187	3.38	0.005
	R × RH	6	0.02019	0.00337	6.06	<0.001

Trait	Source of variation	df	SS	MS	F	P
	LI × RH	4	0.01170	0.00292	5.27	<0.001
	R × LI × RH	12	0.02395	0.00200	3.60	<0.001
	Error	72	0.03995	0.00055	–	–
	Total	107	0.18211	–	–	–
**Stem dry weight (g)**	Rootstock (R)	3	11.8993	3.9664	1787.23	<0.001
	Light intensity (LI)	2	0.0337	0.0168	7.59	0.001
	Relative humidity (RH)	2	0.3368	0.1684	75.88	<0.001
	R × LI	6	0.1009	0.0168	7.58	<0.001
	R × RH	6	0.1381	0.0230	10.37	<0.001
	LI × RH	4	0.0315	0.0079	3.55	0.011
	R × LI × RH	12	0.1963	0.0164	7.37	<0.001
	Error	72	0.1598	0.0022	–	–
	Total	107	12.8964	–	–	–
**Leaf dry weight (g)**	Rootstock (R)	3	1.293	0.431	23.44	<0.001
	Light intensity (LI)	2	0.074	0.037	2.02	0.140
	Relative humidity (RH)	2	2.326	1.163	63.26	<0.001
	R × LI	6	0.763	0.127	6.91	<0.001
	R × RH	6	1.078	0.180	9.77	<0.001
	LI × RH	4	0.123	0.031	1.68	0.165
	R × LI × RH	12	1.509	0.126	6.84	<0.001
	Error	72	1.323	0.018	–	–
	Total	107	8.489	–	–	–

df, degrees of freedom; SS, sum of squares; MS, mean square (SS/df); F, F-ratio (MS/MS of error); P, probability. Three-way ANOVA with rootstock, pre-grafting light intensity and healing relative humidity as fixed factors (n = 3 replicates per treatment combination).

Table S2. Complete analysis of variance for gas exchange and chlorophyll fluorescence parameters of grafted tomato seedlings.

Trait	Source of variation	df	SS	MS	F	P
**Net photosynthetic rate**	Rootstock (R)	3	4809.96	1603.32	961.06	<0.001
	Light intensity (LI)	2	48.43	24.22	14.52	<0.001
	Relative humidity (RH)	2	229.15	114.58	68.68	<0.001
	R × LI	6	96.84	16.14	9.67	<0.001
	R × RH	6	72.10	12.02	7.20	<0.001
	LI × RH	4	100.22	25.06	15.02	<0.001
	R × LI × RH	12	74.42	6.20	3.72	<0.001
	Error	72	120.12	1.67	–	–
	Total	107	5551.25	–	–	–
**Stomatal conductance**	Rootstock (R)	3	0.3906	0.1302	44.51	<0.001
	Light intensity (LI)	2	0.0272	0.0136	4.65	0.013
	Relative humidity (RH)	2	0.0935	0.0467	15.98	<0.001
	R × LI	6	0.1109	0.0185	6.32	<0.001
	R × RH	6	0.1828	0.0305	10.42	<0.001
	LI × RH	4	0.1893	0.0473	16.18	<0.001
	R × LI × RH	12	0.1866	0.0156	5.32	<0.001
	Error	72	0.2106	0.0029	–	–
	Total	107	1.3916	–	–	–
**Transpiration rate**	Rootstock (R)	3	27.29	9.10	46.23	<0.001
	Light intensity (LI)	2	1.39	0.70	3.54	0.034
	Relative humidity (RH)	2	3.08	1.54	7.84	<0.001
	R × LI	6	6.18	1.03	5.24	<0.001
	R × RH	6	19.82	3.30	16.79	<0.001
	LI × RH	4	8.31	2.08	10.56	<0.001
	R × LI × RH	12	13.82	1.15	5.85	<0.001
	Error	72	14.17	0.20	–	–
	Total	107	94.06	–	–	–
**Fv/Fm**	Rootstock (R)	3	0.22486	0.07495	368.43	<0.001
	Light intensity (LI)	2	0.01059	0.00529	26.03	<0.001
	Relative humidity (RH)	2	0.09242	0.04621	227.14	<0.001
	R × LI	6	0.06709	0.01118	54.96	<0.001
	R × RH	6	0.05570	0.00928	45.63	<0.001
	LI × RH	4	0.07711	0.01928	94.75	<0.001
	R × LI × RH	12	0.35691	0.02974	146.20	<0.001
	Error	72	0.01465	0.00020	–	–
	Total	107	0.89932	–	–	–

df, degrees of freedom; SS, sum of squares; MS, mean square (SS/df); F, F-ratio (MS/MS of error); P, probability. Three-way ANOVA with rootstock, pre-grafting light intensity and healing relative humidity as fixed factors (n = 3 replicates per treatment combination).

Table S3. Growth and biomass traits of Jalila F1 tomato seedlings grafted onto four rootstocks under nine combinations of pre-grafting light intensity and healing relative humidity (RH).

Rootstock	Light (μmol m^{-2} s^{-1})	RH (%)	Plant height (cm)	Root length (cm)	Leaf area (cm^2)	Stem diameter (mm)	No. of true leaves	Root dry weight (g)	Stem dry weight (g)	Leaf dry weight (g)
**Auroch**	150	60	21.17 ± 0.44 bc	29.17 ± 0.93 ab	244.79 ± 11.45 b	5.07 ± 0.05 abc	5.33 ± 0.33 bc	0.101 ± 0.016 ab	1.002 ± 0.042 cde	1.007 ± 0.091 bc
	150	75	26.00 ± 0.58 a	21.33 ± 0.44 c	311.71 ± 4.66 a	4.49 ± 0.23 cd	6.00 ± 0.00 ab	0.082 ± 0.013 abc	1.170 ± 0.074 a	1.411 ± 0.180 a
	150	90	18.83 ± 0.44 cd	24.67 ± 1.59 bc	173.10 ± 13.37 cd	4.61 ± 0.21 bcd	5.67 ± 0.33 abc	0.038 ± 0.017 cd	0.926 ± 0.021 de	0.554 ± 0.018 d
	250	60	21.83 ± 1.36 b	34.67 ± 4.84 a	163.47 ± 13.36 cd	4.57 ± 0.28 cd	5.00 ± 0.00 bc	0.088 ± 0.026 abc	1.017 ± 0.014 bcd	0.776 ± 0.018 cd
	250	75	25.67 ± 0.93 a	30.33 ± 0.88 ab	232.73 ± 3.32 b	5.67 ± 0.08 a	5.67 ± 0.33 abc	0.131 ± 0.025 ab	1.176 ± 0.028 a	1.323 ± 0.158 ab
	250	90	16.33 ± 0.17 d	24.67 ± 1.76 bc	141.92 ± 11.80 d	4.24 ± 0.07 d	4.67 ± 0.33 c	0.014 ± 0.011 d	0.882 ± 0.013 e	0.557 ± 0.041 d
	350	60	20.67 ± 0.44 bc	27.67 ± 0.88 abc	206.71 ± 7.88 bc	5.13 ± 0.14 abc	5.67 ± 0.33 abc	0.072 ± 0.029 bcd	1.029 ± 0.044 bcd	0.990 ± 0.065 bc
	350	75	22.50 ± 0.29 b	26.67 ± 0.88 bc	173.07 ± 8.51 cd	5.06 ± 0.01 abcd	5.67 ± 0.33 abc	0.114 ± 0.010 ab	1.100 ± 0.022 abc	1.064 ± 0.057 abc
	350	90	26.00 ± 0.58 a	29.33 ± 0.88 ab	216.55 ± 1.33 bc	5.43 ± 0.21 ab	6.67 ± 0.33 a	0.137 ± 0.006 a	1.139 ± 0.018 ab	1.208 ± 0.012 ab
**Ficus**	150	60	19.83 ± 1.17 ab	24.67 ± 1.45 ab	277.72 ± 5.05 abc	5.07 ± 0.16 b	6.33 ± 0.33 a	0.107 ± 0.006 abc	0.373 ± 0.012 abc	1.160 ± 0.010 ab
	150	75	19.67 ± 0.67 ab	23.50 ± 1.26 b	250.24 ± 11.25 abcd	5.46 ± 0.12 b	6.00 ± 0.00 ab	0.059 ± 0.016 c	0.264 ± 0.051 cd	0.729 ± 0.140 c
	150	90	18.67 ± 0.33 b	22.67 ± 0.33 b	233.46 ± 9.82 bcd	5.64 ± 0.42 b	6.00 ± 0.00 ab	0.082 ± 0.004 bc	0.337 ± 0.009 bcd	0.922 ± 0.004 bc
	250	60	21.50 ± 0.58 ab	27.33 ± 0.88 ab	284.11 ± 2.31 ab	5.27 ± 0.14 b	6.33 ± 0.33 a	0.118 ± 0.005 abc	0.367 ± 0.007 abc	1.101 ± 0.044 ab
	250	75	21.83 ± 1.01 a	23.50 ± 0.76 b	251.87 ± 9.52 abcd	6.67 ± 0.13 a	6.00 ± 0.00 ab	0.128 ± 0.007 ab	0.471 ± 0.026 a	1.326 ± 0.038 a
	250	90	22.00 ± 1.26 a	30.83 ± 3.22 a	297.63 ± 26.41 a	5.57 ± 0.23 b	6.00 ± 0.00 ab	0.078 ± 0.005 bc	0.384 ± 0.039 abc	1.115 ± 0.127 ab
	350	60	21.50 ± 0.76 ab	20.33 ± 0.33 b	249.05 ± 6.10 abcd	4.95 ± 0.14 b	6.00 ± 0.00 ab	0.162 ± 0.013 a	0.432 ± 0.020 ab	1.302 ± 0.123 a
	350	75	21.83 ± 0.60 a	22.83 ± 1.30 b	228.06 ± 11.63 cd	5.02 ± 0.09 b	6.00 ± 0.00 ab	0.123 ± 0.005 ab	0.428 ± 0.018 ab	1.201 ± 0.027 ab
	350	90	15.50 ± 0.76 c	22.00 ± 0.58 b	214.42 ± 1.64 d	5.01 ± 0.05 b	5.00 ± 0.00 b	0.088 ± 0.002 bc	0.236 ± 0.004 d	0.874 ± 0.006 bc
**Goldrake**	150	60	16.67 ± 0.73 cd	29.00 ± 1.00 a	181.43 ± 15.94 bc	5.43 ± 0.16 ab	5.00 ± 0.00 ab	0.132 ± 0.033 a	0.289 ± 0.018 cd	0.977 ± 0.053 ab
	150	75	17.33 ± 0.17 cd	27.33 ± 2.33 ab	216.22 ± 9.34 ab	5.99 ± 0.14 a	6.00 ± 0.00 a	0.133 ± 0.009 a	0.356 ± 0.013 bc	1.136 ± 0.045 ab
	150	90	12.83 ± 0.60 e	21.83 ± 1.09 bc	120.27 ± 31.30 d	3.85 ± 0.39 cd	4.67 ± 0.33 b	0.055 ± 0.011 bc	0.151 ± 0.038 e	0.482 ± 0.162 c
	250	60	20.33 ± 0.67 b	25.00 ± 0.58 abc	237.23 ± 10.00 a	5.64 ± 0.15 a	6.00 ± 0.00 a	0.127 ± 0.015 a	0.409 ± 0.002 abc	1.280 ± 0.066 a
	250	75	24.17 ± 0.44 a	24.67 ± 1.20 abc	211.82 ± 7.51 ab	5.83 ± 0.03 a	6.00 ± 0.00 a	0.154 ± 0.008 a	0.500 ± 0.013 a	1.106 ± 0.019 ab
	250	90	15.67 ± 0.93 de	22.00 ± 2.52 abc	153.24 ± 15.14 cd	4.67 ± 0.11 bc	5.33 ± 0.33 ab	0.032 ± 0.005 c	0.225 ± 0.027 de	0.824 ± 0.017 bc
	350	60	21.33 ± 0.33 ab	22.67 ± 1.20 abc	238.17 ± 16.89 a	5.43 ± 0.18 ab	5.67 ± 0.33 ab	0.142 ± 0.011 a	0.422 ± 0.024 ab	1.226 ± 0.051 a
	350	75	19.33 ± 0.33 bc	23.00 ± 0.58 abc	230.82 ± 16.66 ab	5.71 ± 0.29 a	5.67 ± 0.33 ab	0.098 ± 0.013 ab	0.320 ± 0.035 bcd	1.229 ± 0.110 a
	350	90	15.50 ± 0.50 de	18.67 ± 0.88 c	138.55 ± 24.05 cd	3.75 ± 0.18 d	5.00 ± 0.00 ab	0.029 ± 0.016 c	0.127 ± 0.026 e	0.510 ± 0.094 c
**TOR23901**	150	60	16.50 ± 0.29 a	27.67 ± 0.67 ab	130.50 ± 4.22 a	4.35 ± 0.06 a	5.00 ± 0.00 a	0.108 ± 0.006 a	0.223 ± 0.024 ab	0.929 ± 0.061 ab
	150	75	16.83 ± 0.44 a	30.00 ± 2.08 a	122.70 ± 4.16 ab	4.10 ± 0.02 a	5.00 ± 0.00 a	0.124 ± 0.006 a	0.276 ± 0.023 a	0.967 ± 0.030 a
	150	90	15.33 ± 1.01 ab	23.17 ± 1.64 ab	98.72 ± 8.19 ab	3.75 ± 0.22 a	5.00 ± 0.00 a	0.078 ± 0.013 a	0.197 ± 0.039 ab	0.752 ± 0.083 ab
	250	60	17.33 ± 0.60 a	29.00 ± 1.00 ab	121.99 ± 7.34 ab	3.88 ± 0.08 a	5.00 ± 0.00 a	0.112 ± 0.006 a	0.240 ± 0.006 ab	0.815 ± 0.029 ab
	250	75	16.33 ± 0.33 a	27.67 ± 2.33 ab	113.45 ± 7.86 ab	4.20 ± 0.10 a	5.00 ± 0.00 a	0.136 ± 0.018 a	0.238 ± 0.012 ab	0.840 ± 0.065 ab
	250	90	13.33 ± 0.60 bc	27.00 ± 0.58 ab	74.14 ± 4.72 b	3.66 ± 0.26 a	5.00 ± 0.00 a	0.075 ± 0.009 a	0.165 ± 0.016 ab	0.645 ± 0.058 ab
	350	60	13.33 ± 0.33 bc	24.33 ± 1.76 ab	83.72 ± 7.77 ab	3.79 ± 0.21 a	4.33 ± 0.33 a	0.121 ± 0.008 a	0.170 ± 0.016 ab	0.756 ± 0.060 ab

Rootstock	Light (μmol m⁻² s⁻¹)	RH (%)	Plant height (cm)	Root length (cm)	Leaf area (cm²)	Stem diameter (mm)	No. of true leaves	Root dry weight (g)	Stem dry weight (g)	Leaf dry weight (g)
	350	75	16.33 ± 0.17 a	22.00 ± 1.00 b	89.84 ± 3.94 ab	4.25 ± 0.11 a	4.67 ± 0.33 a	0.129 ± 0.004 a	0.211 ± 0.021 ab	0.743 ± 0.048 ab
	350	90	12.33 ± 0.33 c	24.67 ± 1.20 ab	70.25 ± 4.75 b	3.64 ± 0.16 a	4.67 ± 0.33 a	0.076 ± 0.009 a	0.129 ± 0.018 b	0.576 ± 0.064 b

Values are means ± SE (n = 3 replicates, one plant measured per replicate). Within each rootstock, means in a column followed by different lowercase letters differ significantly among light intensity × relative humidity combinations according to Tukey's HSD test ($p < 0.05$), based on the pooled error of the three-way ANOVA. Letters should not be compared across rootstocks.

Table S4. Gas exchange and chlorophyll fluorescence parameters of Jalila F1 tomato seedlings grafted onto four rootstocks under nine combinations of pre-grafting light intensity and healing relative humidity (RH).

Rootstock	Light (μmol m^{-2} s^{-1})	RH (%)	Pn (μmol CO_2 m^{-2} s^{-1})	gs (mol H_2O m^{-2} s^{-1})	E (mmol H_2O m^{-2} s^{-1})	Fv/Fm	Y(II)	NPQ	ETR (μmol e^- m^{-2} s^{-1})
**Auroch**	150	60	7.52 ± 0.40 a	0.331 ± 0.011 a	3.29 ± 0.11 a	0.737 ± 0.007 bc	0.147 ± 0.006 bc	0.426 ± 0.013 de	20.70 ± 0.85 bc
	150	75	6.43 ± 0.19 abc	0.291 ± 0.042 ab	2.81 ± 0.27 ab	0.767 ± 0.003 ab	0.149 ± 0.004 bc	0.584 ± 0.006 ab	21.03 ± 0.50 b
	150	90	3.71 ± 0.39 bcd	0.187 ± 0.051 bc	2.05 ± 0.41 bc	0.760 ± 0.010 ab	0.136 ± 0.007 cd	0.636 ± 0.011 a	19.10 ± 0.96 bc
	250	60	2.68 ± 0.29 d	0.150 ± 0.023 bc	1.85 ± 0.19 bc	0.793 ± 0.003 a	0.100 ± 0.004 e	0.517 ± 0.041 bcd	14.00 ± 0.61 d
	250	75	9.09 ± 0.89 a	0.357 ± 0.011 a	3.39 ± 0.07 a	0.737 ± 0.003 bc	0.187 ± 0.003 a	0.513 ± 0.004 bcd	26.13 ± 0.67 a
	250	90	2.53 ± 0.10 d	0.118 ± 0.004 c	1.53 ± 0.05 c	0.703 ± 0.009 c	0.163 ± 0.002 ab	0.527 ± 0.030 bc	22.43 ± 0.37 b
	350	60	3.51 ± 0.50 bcd	0.180 ± 0.079 bc	2.01 ± 0.66 bc	0.717 ± 0.007 c	0.103 ± 0.005 e	0.380 ± 0.009 e	14.47 ± 0.66 d
	350	75	6.66 ± 0.15 ab	0.243 ± 0.039 abc	2.58 ± 0.29 abc	0.770 ± 0.006 ab	0.121 ± 0.008 de	0.494 ± 0.005 bcd	17.07 ± 1.04 cd
	350	90	3.11 ± 0.40 cd	0.112 ± 0.013 c	1.47 ± 0.14 c	0.757 ± 0.007 ab	0.157 ± 0.006 bc	0.480 ± 0.014 cd	22.03 ± 0.75 b
**Ficus**	150	60	2.14 ± 0.40 c	0.206 ± 0.040 abc	2.40 ± 0.36 ab	0.697 ± 0.019 cd	0.063 ± 0.005 ab	0.697 ± 0.033 b	8.07 ± 0.55 b
	150	75	4.41 ± 0.55 abc	0.224 ± 0.044 ab	2.58 ± 0.40 ab	0.670 ± 0.006 de	0.067 ± 0.009 ab	0.620 ± 0.036 b	9.73 ± 0.96 b
	150	90	3.66 ± 0.31 abc	0.131 ± 0.004 abc	1.78 ± 0.04 bc	0.657 ± 0.009 e	0.052 ± 0.001 b	0.281 ± 0.006 c	8.13 ± 0.44 b
	250	60	2.65 ± 0.37 bc	0.142 ± 0.011 abc	1.83 ± 0.13 bc	0.697 ± 0.003 cd	0.054 ± 0.002 b	0.300 ± 0.002 c	8.90 ± 0.40 b
	250	75	5.62 ± 0.25 ab	0.117 ± 0.004 bc	1.65 ± 0.05 bc	0.767 ± 0.013 a	0.102 ± 0.004 a	0.333 ± 0.014 c	13.73 ± 0.95 a
	250	90	2.41 ± 0.56 bc	0.072 ± 0.016 c	1.08 ± 0.22 c	0.647 ± 0.003 e	0.050 ± 0.025 b	0.734 ± 0.004 ab	11.13 ± 0.59 ab
	350	60	1.78 ± 0.49 c	0.070 ± 0.014 c	1.05 ± 0.18 c	0.720 ± 0.006 bc	0.054 ± 0.003 b	0.395 ± 0.019 c	13.07 ± 0.93 a
	350	75	5.63 ± 0.18 ab	0.089 ± 0.004 bc	0.69 ± 0.35 c	0.773 ± 0.003 a	0.056 ± 0.003 ab	0.851 ± 0.039 a	8.10 ± 0.25 b
	350	90	6.44 ± 0.25 a	0.270 ± 0.015 a	3.09 ± 0.14 a	0.753 ± 0.009 ab	0.061 ± 0.001 ab	0.828 ± 0.023 a	8.60 ± 0.10 b
**Goldrake**	150	60	3.73 ± 0.46 b	0.086 ± 0.006 d	1.23 ± 0.07 c	0.367 ± 0.022 d	0.141 ± 0.014 bc	0.519 ± 0.014 a	11.97 ± 2.31 c
	150	75	9.24 ± 0.07 a	0.107 ± 0.028 cd	0.97 ± 0.03 c	0.683 ± 0.009 bc	0.107 ± 0.001 cd	0.473 ± 0.026 abc	14.57 ± 0.55 c
	150	90	2.31 ± 0.29 b	0.252 ± 0.031 b	2.79 ± 0.23 a	0.670 ± 0.006 bc	0.202 ± 0.018 a	0.391 ± 0.025 cd	28.53 ± 2.55 a
	250	60	5.11 ± 0.37 b	0.043 ± 0.009 d	0.64 ± 0.12 c	0.657 ± 0.009 c	0.088 ± 0.004 d	0.506 ± 0.005 a	12.43 ± 0.59 c
	250	75	12.28 ± 0.46 a	0.467 ± 0.073 a	1.44 ± 0.33 bc	0.697 ± 0.009 b	0.179 ± 0.002 ab	0.399 ± 0.022 bcd	26.20 ± 0.35 a
	250	90	4.61 ± 0.44 b	0.241 ± 0.064 bc	2.59 ± 0.47 ab	0.663 ± 0.009 bc	0.140 ± 0.006 bc	0.352 ± 0.013 d	21.87 ± 0.23 ab
	350	60	2.41 ± 0.29 b	0.109 ± 0.023 cd	1.46 ± 0.23 bc	0.740 ± 0.006 a	0.096 ± 0.006 cd	0.493 ± 0.006 ab	13.63 ± 0.76 c
	350	75	3.70 ± 1.39 b	0.113 ± 0.031 bcd	1.48 ± 0.33 bc	0.770 ± 0.006 a	0.117 ± 0.013 cd	0.522 ± 0.029 a	16.43 ± 1.83 bc
	350	90	2.06 ± 1.25 b	0.232 ± 0.056 bc	2.55 ± 0.42 ab	0.360 ± 0.000 d	0.120 ± 0.010 cd	0.323 ± 0.023 d	22.17 ± 0.19 ab
**TOR23901**	150	60	18.78 ± 0.08 b	0.015 ± 0.000 b	0.27 ± 0.00 b	0.733 ± 0.009 ab	0.096 ± 0.004 b	0.484 ± 0.027 abc	15.33 ± 1.33 abc
	150	75	20.37 ± 1.41 b	0.043 ± 0.013 ab	0.71 ± 0.19 b	0.723 ± 0.007 bc	0.098 ± 0.011 b	0.564 ± 0.045 a	13.00 ± 1.15 bcd
	150	90	20.82 ± 0.82 b	0.067 ± 0.009 ab	1.10 ± 0.14 b	0.717 ± 0.003 bc	0.090 ± 0.010 bc	0.476 ± 0.007 abc	15.83 ± 1.86 ab
	250	60	20.35 ± 0.89 b	0.036 ± 0.004 b	0.64 ± 0.07 b	0.660 ± 0.010 d	0.072 ± 0.003 bc	0.421 ± 0.020 c	10.20 ± 0.40 d
	250	75	25.25 ± 0.80 a	0.182 ± 0.034 a	2.60 ± 0.35 a	0.767 ± 0.003 a	0.091 ± 0.003 bc	0.465 ± 0.008 abc	12.03 ± 1.11 bcd
	250	90	18.44 ± 0.54 b	0.040 ± 0.002 b	0.71 ± 0.03 b	0.663 ± 0.009 d	0.087 ± 0.003 bc	0.452 ± 0.010 bc	12.77 ± 0.55 bcd

Rootstock	Light ($\mu mol\ m^{-2}\ s^{-1}$)	RH (%)	Pn ($\mu mol\ CO_2\ m^{-2}\ s^{-1}$)	gs ($mol\ H_2O\ m^{-2}\ s^{-1}$)	E ($mmol\ H_2O\ m^{-2}\ s^{-1}$)	Fv/Fm	Y(II)	NPQ	ETR ($\mu mol\ e^-\ m^{-2}\ s^{-1}$)
	350	60	18.57 ± 0.14 b	0.036 ± 0.001 b	0.65 ± 0.01 b	0.687 ± 0.003 cd	0.082 ± 0.005 bc	0.432 ± 0.011 c	10.30 ± 0.20 cd
	350	75	17.89 ± 1.19 b	0.032 ± 0.001 b	0.57 ± 0.02 b	0.707 ± 0.007 bc	0.067 ± 0.002 c	0.417 ± 0.007 c	8.67 ± 0.86 d
	350	90	19.68 ± 2.58 b	0.063 ± 0.015 ab	1.05 ± 0.23 b	0.657 ± 0.003 d	0.161 ± 0.004 a	0.539 ± 0.008 ab	18.80 ± 0.71 a

Values are means ± SE (n = 3 replicates, one plant measured per replicate). Within each rootstock, means in a column followed by different lowercase letters differ significantly among light intensity × relative humidity combinations according to Tukey's HSD test ($p < 0.05$), based on the pooled error of the three-way ANOVA. Letters should not be compared across rootstocks. For Y(II), NPQ and ETR, letters correspond to those shown in Figures 4–6 (Tukey's HSD test, $p < 0.05$, performed in XLSTAT). Pn, net photosynthetic rate; gs, stomatal conductance; E, transpiration rate; Fv/Fm, maximum quantum efficiency of PSII; Y(II), effective quantum yield of PSII; NPQ, non-photochemical quenching; ETR, electron transport rate.
